# Reporting of analyses from randomized controlled trials with multiple arms: a systematic review

**DOI:** 10.1186/1741-7015-11-84

**Published:** 2013-03-27

**Authors:** Gabriel Baron, Elodie Perrodeau, Isabelle Boutron, Philippe Ravaud

**Affiliations:** 1 INSERM, Paris, U738, France; 2 AP-HP (Assistance Publique des Hôpitaux de Paris), Hôpital Hôtel Dieu, Centre d’Epidémiologie Clinique, Paris, France; 3 Université Paris Descartes, Faculté de Médecine, Paris, France; 4 Centre de Médecine Fondée sur les Preuves (EHESP, HAS, INSERM, AP-HP), Paris, France; 5 Department of Epidemiology, Columbia University Mailman School of Public Health, New York, NY, USA

**Keywords:** Systematic review, Randomized controlled trials, Multiple arms, Reporting of analyses

## Abstract

**Background:**

Multiple-arm randomized trials can be more complex in their design, data analysis, and result reporting than two-arm trials. We conducted a systematic review to assess the reporting of analyses in reports of randomized controlled trials (RCTs) with multiple arms.

**Methods:**

The literature in the MEDLINE database was searched for reports of RCTs with multiple arms published in 2009 in the core clinical journals. Two reviewers extracted data using a standardized extraction form.

**Results:**

In total, 298 reports were identified. Descriptions of the baseline characteristics and outcomes per group were missing in 45 reports (15.1%) and 48 reports (16.1%), respectively. More than half of the articles (n = 171, 57.4%) reported that a planned global test comparison was used (that is, assessment of the global differences between all groups), but 67 (39.2%) of these 171 articles did not report details of the planned analysis. Of the 116 articles reporting a global comparison test, 12 (10.3%) did not report the analysis as planned. In all, 60% of publications (n = 180) described planned pairwise test comparisons (that is, assessment of the difference between two groups), but 20 of these 180 articles (11.1%) did not report the pairwise test comparisons. Of the 204 articles reporting pairwise test comparisons, the comparisons were not planned for 44 (21.6%) of them. Less than half the reports (n = 137; 46%) provided baseline and outcome data per arm and reported the analysis as planned.

**Conclusions:**

Our findings highlight discrepancies between the planning and reporting of analyses in reports of multiple-arm trials.

## Background

Randomized controlled trials (RCTs) with multiple arms are sometimes considered an attractive way of optimizing resources and simultaneously testing various treatment strategies [1-4]. For instance, multiple-arm trials can involve increasing doses of an experimental treatment, cumulative combination therapies, or multiple independent treatments, which can allows testing of the efficacy of new treatments to be carried out more rapidly and more directly [5]. Such trials provide more information than two-arm trials can provide [6]. Multiple-arm randomized trials are becoming increasingly common, with a quarter of randomized trials having more than two intervention groups [7].

However, because of the number of arms, such trials can be more complex in design, data analysis and result reporting compared with two-arm trials [2,8,9]. Complications of such trials are directly related to the number of arms and the number of possible comparisons. For instance, in an RCT with three arms, there are seven theoretically possible comparisons [6]. The complications of such trials include: defining *a priori* which comparisons are of primary interest; the possibility of performing global comparison tests (that is, assessing global differences between all arms) and/or pairwise comparison tests (that is, assessing differences of 2 arms), of pooling data for two or more arms, of reporting selective comparisons (for example, only statistically significant comparisons) or *post hoc* comparisons (for example, comparisons that were not planned in the protocol), or of using a multiple comparison adjustment procedure for controlling type I error rate, which influences sample-size calculation and statistical analysis; and the necessity of having sufficient details of the primary outcomes per group for future meta-analyses. To our knowledge, no systematic review has compared the planned comparisons (as described in reports) and the reported comparisons from multiple-arm trials. We aimed to appraise the reporting of analyses from RCTs with multiple arms by examining a sample of reports of results of such trials published in core clinical journals.

## Methods

### Search strategy

We searched MEDLINE (via PubMed) to identify reports of RCTs indexed between January and December 2009, which were published in the core clinical journals defined by the US National Library of Medicine and the National Institutes of Health (a subset of 119 widely read journals published in English, covering all specialties of clinical medicine and public-health sciences, and including all major medical journals, which was previously known as the Abridged Index Medicus and is available at http://www.nlm.nih.gov/bsd/aim.html). The search strategy used the following limits: ‘randomized controlled trial’, ‘publication date from 2009/01/01 to 2009/12/31’ and ‘core clinical journals’. The date of search was 13 January 2010.

### Eligibility criteria and screening process

One of the researchers (GB) screened the titles and abstracts of retrieved articles to identify relevant articles, then obtained full text of the relevant articles, and assessed the full text to determine whether the article met the inclusion criteria. The help of a second reviewer (IB or EP) was requested if needed. We considered only articles that were the first report of the trial results. We excluded sub-studies of an original publication (for example, follow-up study, trial extension, ancillary study, *post hoc* analyses, exploratory analyses, secondary analyses, reanalysis of a trial, pooled analyses of trials).

### Data collection

A standardized data-extraction form (available from the corresponding author) was generated from a review of the literature and *a priori* discussion. Before data extraction, the form was tested independently, as a calibration exercise, by two of the authors (GB, EP) on a separate random set of 20 articles. The ratings were reviewed and any disagreements were resolved by consensus.

Following this, the two reviewers, who were not blinded to the journal name, authors, author affiliations, or funding sources, retrieved and extracted data from published articles. A random sample of 30 articles was reviewed for quality assurance. Inter-observer agreement in extracting data was good: the median kappa value for items was 0.68 (range 0.30 to 1.00) (see Additional file 1). In cases of uncertainty regarding a particular article, items with poor agreement, or items related to the design of the trial, the data were independently checked by the second reader, and discrepancies were resolved by discussion.

### Data collection

We extracted data related to the general characteristics of the study: number of randomized groups, study design, medical area, nature of intervention group(s), number of centers, total number of randomized participants, randomization design, funding sources, and whether the trial was registered. We also extracted methodological items: definition of the study hypothesis (the comparisons planned in the Methods section), baseline characteristics and outcomes reported per group (details that would allow for future meta-analyses), sample-size calculation reported, sample-size calculation taken into account in the multiple-arm design (either by a global sample-size calculation or by an adjustment method used for multiple testing), planning or use of an adjustment method for statistical comparisons (either for sample-size calculation or for statistical analysis), and whether the title identified the trial as a multiple-arm trial. We also systematically assessed selective reporting by comparing the planned comparisons (that is, the comparisons reported in the Methods section) and reported comparisons (the comparisons reported in the Results section) for global comparison tests (which globally assess differences between all groups), pairwise comparison tests (which compare data between two groups); and pooled group analyses (which assess combined data for two or more groups).

### Statistical analysis

Because we chose a convenience sample of RCTs, we did not calculate a required sample size. Our planned analysis was descriptive, and was stratified by study design (parallel-arm, factorial, crossover). Categorical variables are presented as frequencies and percentages, and quantitative variables are presented as median (with 10th and 90th percentiles). We specifically investigated comparisons that were reported as planned but were not performed, which could suggest selective reporting. We also investigated reported comparisons that were not planned, which could suggest *post hoc* comparisons. Data analysis involved use of the software programs SAS (version 9.3 for Windows; SAS Institute, Cary, NC, USA) and R (version 2.15.1; R Foundation for Statistical Computing, Vienna, Austria).

## Results

### Selection of articles

**Figure 1:**
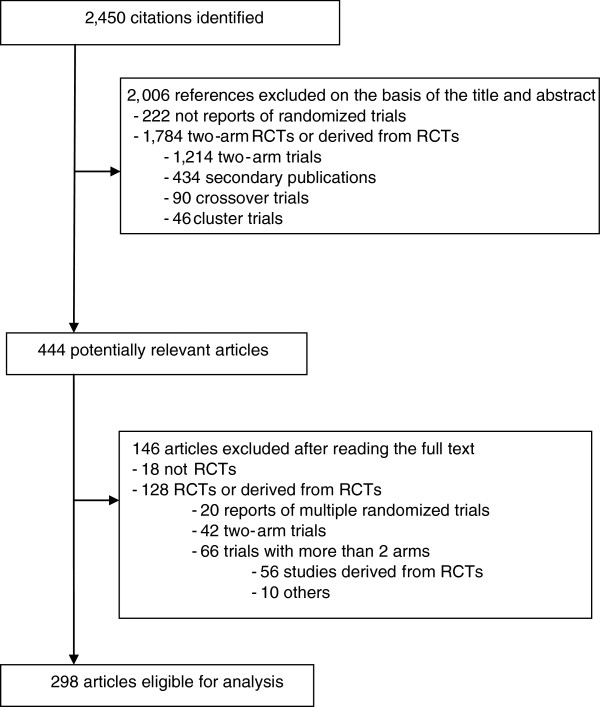
Study screening process.

A flowchart of the selection of articles is shown in Figure 1. Briefly, the electronic search yielded 2,450 citations, and after reading the full text, we selected 298 reports published in 68 journals (see Additional file 2). In all, 48 articles (16.1%) were published in general medical journals and 250 (83.9%) in specialized medical journals.

### Characteristics of multiple-arm trials

The characteristics of the trials are shown in Table 1. Of the 1690 RCTs assessed, the proportion of multiple-arm trials was 17.6% (n = 298; 95% confidence interval 15.8 to 19.5), with 221 (74.2%) having a parallel-group design, 37 a factorial design (12.4%), and 40 (13.4%) a crossover design. The most common intervention was a drug (n = 192, 64.4%), and trials were mostly designed to show superiority (n = 260, 87.3%).

**Table 1:** Characteristics of randomized controlled trials with multiple arms by trial design

	**All trials, n = 298**			**Trial design**		
		**Parallel group, n = 221**	**Parallel group with factorial, n = 37**	**Crossover**^**a**^**, n = 40**	
**No. of study groups**				
3	172 (57.7)	152 (68.8)	0 (0.0)	20 (50.0)
4	84 (28.2)	39 (17.7)	34 (91.9)	11 (27.5)
> 4	42 (14.1)	30 (13.6)	3 (8.1)	9 (22.5)
**Common specialties**				
Anesthesia	54 (18.1)	47 (21.3)	3 (8.1)	4 (10.0
Endocrinology	32 (10.7)	20 (9.1)	5 (13.5)	7 (17.5)
Cardiology	31 (10.4)	25 (11.3)	6 (16.2)	0 (0.0)
Infectious disease	22 (7.4)	20 (9.1)	1 (2.7)	1 (2.5)
Rheumatology	19 (6.4)	18 (8.1)	1 (2.7)	0 (0.0)
**Intervention**				
Drug	192 (64.4)	148 (68.0)	22 (59.5)	22 (55.0)
Surgery or procedure	17 (5.7)	12 (5.4)	1 (2.7)	4 (10.0)
Counseling or lifestyle interventions	47 (15.8)	33 (14.9)	10 (27.0)	4 (10.0)
Equipment or device	26 (8.7)	19 (8.6)	2 (5.4)	5 (12.5)
Others	16 (6.7)	5 (1.7)	2 (0.7)	9 (3.0)
**Study centers**				
Single	80 (26.9)	54 (24.4)	8 (21.6)	18 (5.0)
Multiple	141 (47.3)	110 (49.8)	24 (64.9)	7 (13.5)
Not reported	77 (25.8)	57 (25.8)	5 (13.5)	15 (37.5)
**No. of randomized patients per:**				
Trial, median (10th to 90th percentile)	136 (25 to 800)	148 (45 to 650)	468 (120 to 1653)	21 (12 to 61)
Arm, median (10th to 90th percentile)	39 (7 to 228)	43 (12 to 204)	117 (30 to 413)	5 (3 to 16)
**Cluster trial**	13 (4.4)	8 (3.6)	3 (8.1)	2 (5.0)
**Trial type**				
To show superiority	260 (87.3)	195 (88.8)	33 (89.2)	32 (80.0)
To show non-inferiority or equivalence	12 (4.0)	8 (3.6)	4 (10.8)	0 (0.0)
Pharmacokinetic/pharmacodynamic objective	26 (8.7)	18 (8.1)	0 (0.0)	8 (20.0)
**Randomization design**				
Balanced	263 (88.3)	190 (86.0)	33 (89.2)	40 (100.0)
Unbalanced	30 (10.1)	28 (12.7)	2 (5.4)	0 (0.0)
Unclear	5 (1.7)	3 (1.3)	2 (5.4)	0 (0.0)
**Funding source**				
Solely or partially industry	101 (33.9)	77 (34.8)	11 (29.7)	13 (32.5)
Public	118 (39.6)	87 (39.4)	14 (37.8)	17 (42.5)
None	8 (2.7)	6 (2.7)	1 (2.7)	1 (2.5)
Unknown	71 (23.8)	51 (23.1)	11 (29.8)	9 (22.5.0)
**Trial registration reported**	144 (48.3)	109 (49.3)	27 (73.0)	8 (20.0)

^a^ There were 4 of the 40 crossover trials with a factorial design.

The number of arms varied from 3 to 16, being 3 in 172 reports (57.7%), 4 in 84 reports (28.2%) and more than 4 in 42 reports (14.1%). The median number of participants per arm was 39 (10th to 90th percentile 7 to 228). Overall, 80 reports described a single-center trial (26.9%), and 141 a multicenter trial (47.3%). The source of funding was described as solely or partially industry in 101 reports (33.9%) and public in 118 (39.6%). Characteristics were similar across the three trial-design types (Table 1), although some characteristics reflected the specificity of each subgroup (for example, the number of arms was greater for factorial designs, and the number of randomized patients was larger for parallel trials than for crossover trials).

**Figure 2:**
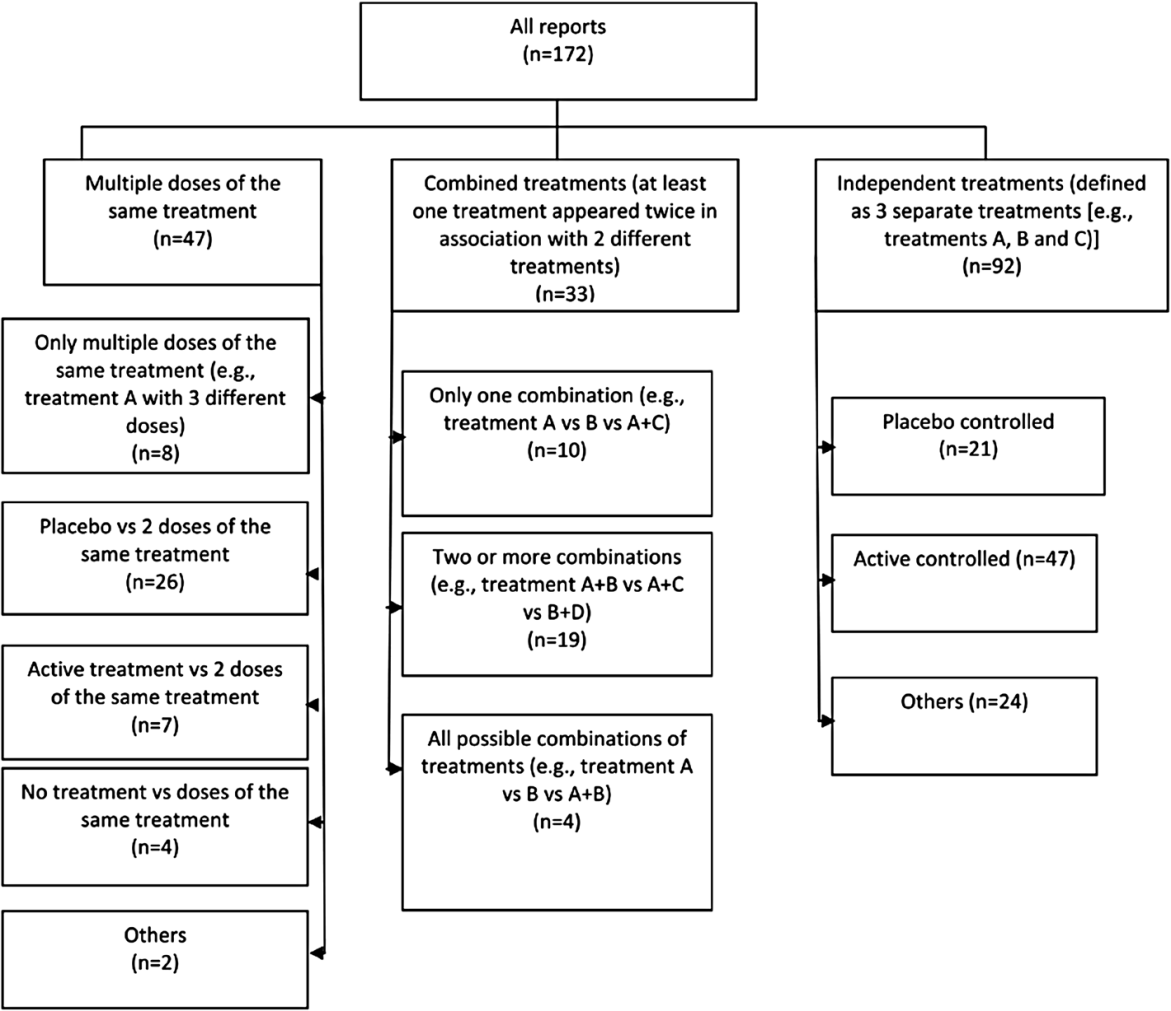
Nature of intervention arms in three-arm randomized controlled trials.

Of the trials with a parallel-group design, excluding those with a factorial design (n = 221, 74.2%), 82 (37.1%) were dose–response trials (use of multiple doses of the same treatment) and 139 (62.9%) compared different treatments. Trials with three arms (n = 172) included several types of interventions and control arms (Figure 2).

### Reporting

Table 2 provides information on the reporting of the results of multiple-arm trials.

**Table 2:** Reporting of randomized controlled trials with multiple arms by trial design

	**All trials, n = 298**	**Trial design**		
		**Parallel group, n = 221**	**Parallel group with factorial, n = 37**	**Crossover (n = 40)**
**Reporting item**				
**Study hypothesis defined**	238 (79.9)	173 (78.3)	33 (89.1)	32 (80.0)
**Baseline characteristics available per group**	253 (84.9)	196 (88.7)	28 (75.7)	29 (72.5)
**Results on outcomes available per group**	250 (83.9)	190 (85.0)	27 (73.0)	33 (82.5)
**Global test comparison**				
Planned	171 (57.4)	127 (57.5)	23 (62.2)	21 (52.5)
Reported	116 (38.9)	84 (38.0)	18 (48.7)	14 (35.0)
**Pairwise test comparisons**				
Planned	180 (60.4)	144 (65.2)	11 (29.7)	25 (62.5)
Reported	204 (68.5)	162 (73.3)	15 (40.5)	27 (67.5)
**Sample-size calculation**				
Reported	210 (70.5)	159 (72.0)	30 (81.1)	21 (52.5)
Taken into account in the multi-arm design	41/210 (19.5)	35/159 (22.0)	5/30 (16.7)	1/21 (4.8)
**Adjustment method used to control type I error due to multiple-arm design**^**a**^	118 (39.6)	89 (40.3)	10 (27.0)	19 (47.50)
**Title identified the study as a multiple-arm trial**	130 (43.6)	101 (45.7)	19 (51.4)	10 (25.0)

^a^ For sample-size calculation or for statistical analysis.

#### Reporting of baseline characteristics and outcomes

The description of baseline characteristics and outcomes per group were missing in 45 (15.1%) and 48 (16.1%), respectively, of the reports investigated. Of the 57 publications describing pooled analyses (19.1%), 17 (28.1%) did not provide results for each randomized group.

#### Planned and reported comparisons

We identified 60 articles (20.1%) that did not define the study hypothesis. More than half of the articles (n = 171, 57.4%) reported that a global comparison test was planned, but 67 (39.2%) did not report the results of the planned analysis. Of the 116 articles reporting a global comparison test, the test was not reported as planned for 12 (10.3%). In all, 60% of publications (n = 180) reported that a pairwise comparison test was planned, but 20 of these (11.1%) did not report a pairwise test comparison. Of the 204 articles reporting pairwise test comparisons, these comparisons were not reported as planned for 44 (21.6%). Less than half of the reports (46%, n = 137) provided baseline and outcome data per group or reported the analysis (global and/or pairwise comparison) as planned.

#### Other elements of reporting

Overall, 70.5% of reports (n = 210) reported a sample-size calculation. The multiple-arm design was taken into account in the sample-size calculation for 41 of 210 reports (19.5%). Of the total of 298 reports, 118 (39.6%) described an adjustment method for multiple statistical comparisons, and 9 (5.0%) of the remaining 180 articles explained why no adjustment was used. Less than half of the trials reports identified the multiple arms in the title (n = 130, 43.6%). For all trials, the reporting of characteristics seemed to be generally poorer for crossover than parallel-group trials, particularly for items concerning sample size (Table 2).

## Discussion

Our findings highlight the inadequate reporting of baseline characteristics and outcomes for arms in multiple-arm RCTs, and the discrepancies between planned and reported comparisons. Moreover, such trials generally had relatively small sample sizes, and showed great variability in the types of intervention and the control arms used.

Multiple-treatment arms are possible sources of multiplicity in an RCT [6,10]. This multiplicity is related to the possibility of performing several pairwise tests to determine the most effective arm. In such a setting, the objectives of the trial must be clear to ensure that these objectives (and only these) are correctly designed and analyzed (for example, global test comparison or not, and which pairwise test comparisons are planned). In 20% of the reports we analyzed, the study hypothesis was not described, which suggests selective reporting. Moreover, bias may be introduced if the decisions on data analysis are driven by the data [11]. For instance, groups receiving different doses of the same intervention could be combined after the data are examined, or only statistically significant pairwise comparisons could be reported. The number of possible comparisons increases greatly in trials with more than three arms, which suggests increased risk of selective reporting. Our results are likely have underestimated any selective outcome reporting bias because we assessed articles and not protocols [12].

Moreover, in our study, some reports did not describe baseline or outcome data for each group (occurred in more than 15% of reports for each scenario). These results are consistent with previous work [13], and are important because reporting data per group is a necessary condition for future meta-analyses.

One of the other methodological difficulties in multiple-arm RCTs concerns the calculation of the sample size, and particularly the necessity for adequate power. Many randomized trials with two parallel arms exhibit inadequate power for revealing differences [14], and sample-size calculation is poorly reported in articles of trials and can be inaccurate [15]. With multiple-arm trials, problems with power and calculation are enhanced, particularly because sample-size calculation depends on the main objective(s) of the trial and thus on the underlying hypotheses that will be tested [1]: whether a global test should be performed or not, and whether (and how many) pairwise comparisons were planned, with statistical adjustment or not.

The question of adjusting for control type I error in multiple-arm trials is a subject of debate [2,3,6,16-18]. Controlling for type I error is not needed when several experimental arms are compared with the control or the standard arms [3], but is necessary when adjusting for *post hoc* comparisons or when the tested hypotheses cannot be prioritized [18]. Reasons for using adjustment or not are often subjective, and should be justified [18].

Our study has several limitations. First, we assessed reports of RCTs and not protocols. This point is particularly important for assessing planned comparisons. We did not assess protocols because of the difficulties in obtaining access to trial protocols [19]. Second, the methods may have been pre-specified but not reported in the articles [20,21]. Third, our results are limited to the core clinical journals defined by the National Library of Medicine, so our findings may not be applicable to journals outside this sample. We chose the core clinical journals because they cover all clinical and public-health areas and all major medical journals. The methodological quality of reports in other journals is unlikely to be better than in these journals.

## Conclusion

The CONSORT (Consolidated Standards of Reporting Trials) group is developing recommendations to help improve the reporting of multiple-arm trials [22,23]. Compared with two-arm RCTs, multiple-arm trials are more complex to design and require more complex analysis, and the results are more complex to report. The design and objectives of the trials have direct consequences for the conduct, analysis of results (for example, planned comparisons, sample-size calculation, adjustment during analysis) and reporting. The specific characteristics of multiple-arm trials and their heterogeneity in objectives, in addition to the usual requirements for reporting the results of RCTs (such as randomization, concealment, and blinding), pose a supplementary challenge for authors reporting the results of multiple-arm trials.

## Competing interests

The authors declare that they have no competing interests.

## Authors’ contributions

GB, IB and PR conceived and designed the study; GB and EP performed the data collection, GP, wrote the first draft of the paper, and EP, IB, and PR critically revised the manuscript for important intellectual content. GB is the guarantor. All authors approved the final version of the manuscript to be published.

## Pre-publication history

The pre-publication history for this paper can be accessed here:

http://www.biomedcentral.com/1741-7015/11/84/prepub

## Supplementary Material

Additional file 1 Reproducibility between reviewers for reporting of items. Click here for file

Additional file 2 List of the 298 articles included in this study. Click here for file
