# Supplementary material for: Reporting of analyses from randomized controlled trials with multiple arms: a systematic review
Source: BMC Med. 2013 Mar 27;11:84. doi: 10.1186/1741-7015-11-84 (PMC3621416; doi:10.1186/1741-7015-11-84)
Supplement: Additional file 1 — Reproducibility between reviewers for reporting of items. [file 1741-7015-11-84-S1.pdf]

Appendix 1. Reproducibility between reviewers for reporting items

	Observed agreement (%)	95% CI	Kappa	95% CI
Study centers*	90.0	73.5-97.9	0.85	0.68-1.00
No. of study groups*	96.7	82.8-99.9	0.95	0.86-1.00
No. of randomized patients*	93.3	77.9-99.3	0.93	0.84-1.00
Cluster trial*	96.7	82.8-99.9	0.93	0.80-1.00
Trial type*	100.0	88.4-100.0	1.00	-
Randomization design*	93.3	77.9-99.2	0.47	-0.13-1.00
Sample size calculation reported**	90.8	87.0-93.9	0.78	0.71-0.86
Funding source*	80.0	61.4-72.3	0.70	0.49-0.91
Trial registration reported*	96.7	82.8-99.9	0.93	0.80-1.00
Title identifies the study as a multiple-arm trial*	96.7	82.8-99.9	0.87	0.62-1.00
Adjustment method to control type I error**	83.0	78.2-87.1	0.64	0.55-0.73
Global analysis planned**	67.9	61.7-72.7	0.30	0.19-0.42
Global analysis reported**	76.8	71.4-81.7	0.42	0.52-0.62
Pairwise comparisons planned**	74.2	68.8-79.1	0.46	0.36-0.56
Pairwise comparisons reported**	81.3	76.4-85.6	0.56	0.46-0.67
Groups pooled**	88.9	84.8-92.2	0.65	0.65-0.76
Baseline characteristics of patients available per group**	84.2	79.5-88.1	0.32	0.19-0.46
Results on outcomes available per group**	86.9	82.5-90.5	0.40	0.25-0.56

95%CI: 95% confidence interval
*n=30
**n=298
