# Supplementary material for: Reporting of analyses from randomized controlled trials with multiple arms: a systematic review
Source: BMC Med. 2013 Mar 27;11:84. doi: 10.1186/1741-7015-11-84 (PMC3621416; doi:10.1186/1741-7015-11-84)
Supplement: Additional file 2 — List of the 298 articles included in this study. [file 1741-7015-11-84-S2.pdf]

Appendix 2. List of the 298 included articles

1. Agarwal-Kozlowski K, Lange AC, Beck H: **Contact-free infrared thermography for assessing effects during acupuncture: a randomized, single-blinded, placebo-controlled crossover clinical trial**. *Anesthesiology* 2009, **111**(3):632-639.
2. Airan-Javia SL, Wolf RL, Wolfe ML, Tadesse M, Mohler E, Reilly MP: **Atheroprotective lipoprotein effects of a niacin-simvastatin combination compared to low- and high-dose simvastatin monotherapy**. *Am Heart J* 2009, **157**(4):687 e681-688.
3. Albrecht A, Kalil RA, Schuch L, Abrahao R, Sant'Anna JR, de Lima G, Nesralla IA: **Randomized study of surgical isolation of the pulmonary veins for correction of permanent atrial fibrillation associated with mitral valve disease**. *J Thorac Cardiovasc Surg* 2009, **138**(2):454-459.
4. Ali J, Al Ahmadi K, Williams JI, Cherry RA: **The standardized live patient and mechanical patient models--their roles in trauma teaching**. *J Trauma* 2009, **66**(1):98-102.
5. Alousi AM, Weisdorf DJ, Logan BR, Bolanos-Meade J, Carter S, Difronzo N, Pasquini M, Goldstein SC, Ho VT, Hayes-Lattin B *et al*: **Etanercept, mycophenolate, denileukin, or pentostatin plus corticosteroids for acute graft-versus-host disease: a randomized phase 2 trial from the Blood and Marrow Transplant Clinical Trials Network**. *Blood* 2009, **114**(3):511-517.
6. Amar SM, Harbeck RJ, Sills M, Silveira LJ, O'Brien H, Nelson HS: **Response to sublingual immunotherapy with grass pollen extract: monotherapy versus combination in a multiallergen extract**. *J Allergy Clin Immunol* 2009, **124**(1):150-156 e151-155.
7. Andrieu G, Roth B, Ousmane L, Castaner M, Petillot P, Vallet B, Villers A, Lebuffe G: **The efficacy of intrathecal morphine with or without clonidine for postoperative analgesia after radical prostatectomy**. *Anesth Analg* 2009, **108**(6):1954-1957.
8. Appukutty J, Shroff PP: **Nasogastric tube insertion using different techniques in anesthetized patients: a prospective, randomized study**. *Anesth Analg* 2009, **109**(3):832-835.
9. Archer DF, Dupont CM, Constantine GD, Pickar JH, Olivier S: **Desvenlafaxine for the treatment of vasomotor symptoms associated with menopause: a double-blind, randomized, placebo-controlled trial of efficacy and safety**. *Am J Obstet Gynecol* 2009, **200**(3):238 e231-238 e210.
10. Archer DF, Seidman L, Constantine GD, Pickar JH, Olivier S: **A double-blind, randomly assigned, placebo-controlled study of desvenlafaxine efficacy and safety for the treatment of vasomotor symptoms associated with menopause**. *Am J Obstet Gynecol* 2009, **200**(2):172 e171-110.
11. Arendt-Nielsen L, Olesen AE, Staahl C, Menzaghi F, Kell S, Wong GY, Drewes AM: **Analgesic efficacy of peripheral kappa-opioid receptor agonist CR665 compared to oxycodone in a multi-modal, multi-tissue experimental human pain model: selective effect on visceral pain**. *Anesthesiology* 2009, **111**(3):616-624.
12. Arroliga AC, Guntupalli KK, Beaver JS, Langholff W, Marino K, Kelly K: **Pharmacokinetics and pharmacodynamics of six epoetin alfa dosing regimens in anemic critically ill patients without acute blood loss**. *Crit Care Med* 2009, **37**(4):1299-1307.

13. Astrup A, Rossner S, Van Gaal L, Rissanen A, Niskanen L, Al Hakim M, Madsen J, Rasmussen MF, Lean ME: **Effects of liraglutide in the treatment of obesity: a randomised, double-blind, placebo-controlled study**. *Lancet* 2009, **374**(9701):1606-1616.
14. Bach-Huynh TG, Nayak B, Loh J, Soldin S, Jonklaas J: **Timing of levothyroxine administration affects serum thyrotropin concentration**. *J Clin Endocrinol Metab* 2009, **94**(10):3905-3912.
15. Banaszewska B, Pawelczyk L, Spaczynski RZ, Duleba AJ: **Comparison of simvastatin and metformin in treatment of polycystic ovary syndrome: prospective randomized trial**. *J Clin Endocrinol Metab* 2009, **94**(12):4938-4945.
16. Bektas F, Eken C, Karadeniz O, Goksu E, Cubuk M, Cete Y: **Intravenous paracetamol or morphine for the treatment of renal colic: a randomized, placebo-controlled trial**. *Ann Emerg Med* 2009, **54**(4):568-574.
17. Bhat KV, Naseeruddin K, Nagalotimath US, Kumar PR, Hegde JS: **Cortical mastoidectomy in quiescent, tubotympanic, chronic otitis media: is it routinely necessary?** *J Laryngol Otol* 2009, **123**(4):383-390.
18. Bhatt T, Pai YC: **Prevention of slip-related backward balance loss: the effect of session intensity and frequency on long-term retention**. *Arch Phys Med Rehabil* 2009, **90**(1):34-42.
19. Bijkerk CJ, de Wit NJ, Muris JW, Whorwell PJ, Knottnerus JA, Hoes AW: **Soluble or insoluble fibre in irritable bowel syndrome in primary care? Randomised placebo controlled trial**. *Bmj* 2009, **339**:b3154.
20. Bingqian L, Peihuan L, Yudong W, Jinxing W, Zhiyong W: **Intraprostatic local anesthesia with periprostatic nerve block for transrectal ultrasound guided prostate biopsy**. *J Urol* 2009, **182**(2):479-483; discussion 483-474.
21. Block SL, Kelsey D, Coury D, Lewis D, Quintana H, Sutton V, Schuh K, Allen AJ, Sumner C: **Once-daily atomoxetine for treating pediatric attention-deficit/hyperactivity disorder: comparison of morning and evening dosing**. *Clin Pediatr (Phila)* 2009, **48**(7):723-733.
22. Bloomfield D, Carlson GL, Sapre A, Tribble D, McKenney JM, Littlejohn TW, 3rd, Sisk CM, Mitchel Y, Pasternak RC: **Efficacy and safety of the cholesteryl ester transfer protein inhibitor anacetrapib as monotherapy and coadministered with atorvastatin in dyslipidemic patients**. *Am Heart J* 2009, **157**(2):352-360 e352.
23. Borer KT, Wuorinen E, Ku K, Burant C: **Appetite responds to changes in meal content, whereas ghrelin, leptin, and insulin track changes in energy availability**. *J Clin Endocrinol Metab* 2009, **94**(7):2290-2298.
24. Bosworth HB, Olsen MK, Dudley T, Orr M, Goldstein MK, Datta SK, McCant F, Gentry P, Simel DL, Oddone EZ: **Patient education and provider decision support to control blood pressure in primary care: a cluster randomized trial**. *Am Heart J* 2009, **157**(3):450-456.
25. Bosworth HB, Olsen MK, Grubber JM, Neary AM, Orr MM, Powers BJ, Adams MB, Svetkey LP, Reed SD, Li Y *et al*: **Two self-management interventions to improve hypertension control: a randomized trial**. *Ann Intern Med* 2009, **151**(10):687-695.
26. Bouvet L, Stoian A, Rimmele T, Allaouchiche B, Chassard D, Boselli E: **Optimal remifentanil dosage for providing excellent intubating conditions when co-administered with a single standard dose of propofol**. *Anaesthesia* 2009, **64**(7):719-726.
27. Bryan AD, Schmiege SJ, Broaddus MR: **HIV risk reduction among detained adolescents: a randomized, controlled trial**. *Pediatrics* 2009, **124**(6):e1180-1188.

28. Buist DS, Anderson ML, Reed SD, Aiello Bowles EJ, Fitzgibbons ED, Gandara JC, Seger D, Newton KM: **Short-term hormone therapy suspension and mammography recall: a randomized trial**. *Ann Intern Med* 2009, **150**(11):752-765.
29. Caballero M, Navarrete P, Prades E, Domenech J, Bernal-Sprekelsen M: **Randomized, placebo-controlled evaluation of chlorobutanol, potassium carbonate, and irrigation in cerumen removal**. *Ann Otol Rhinol Laryngol* 2009, **118**(8):552-555.
30. Cacchio A, Giordano L, Colafarina O, Rompe JD, Tavernese E, Ioppolo F, Flamini S, Spacca G, Santilli V: **Extracorporeal shock-wave therapy compared with surgery for hypertrophic long-bone nonunions**. *J Bone Joint Surg Am* 2009, **91**(11):2589-2597.
31. Cals JW, Butler CC, Hopstaken RM, Hood K, Dinant GJ: **Effect of point of care testing for C reactive protein and training in communication skills on antibiotic use in lower respiratory tract infections: cluster randomised trial**. *Bmj* 2009, **338**:b1374.
32. Candiotti KA, Bergese SD, Bokesch PM, Feldman MA, Wisemandle W, Bekker AY: **Monitored anesthesia care with dexmedetomidine: a prospective, randomized, double-blind, multicenter trial**. *Anesth Analg*, **110**(1):47-56.
33. Canuso CM, Dirks B, Carothers J, Kosik-Gonzalez C, Bossie CA, Zhu Y, Damaraju CV, Kalali AH, Mahmoud R: **Randomized, double-blind, placebo-controlled study of paliperidone extended-release and quetiapine in inpatients with recently exacerbated schizophrenia**. *Am J Psychiatry* 2009, **166**(6):691-701.
34. Carmona-Fonseca J, Alvarez G, Maestre A: **Methemoglobinemia and adverse events in Plasmodium vivax malaria patients associated with high doses of primaquine treatment**. *Am J Trop Med Hyg* 2009, **80**(2):188-193.
35. Cashman KD, Wallace JM, Horigan G, Hill TR, Barnes MS, Lucey AJ, Bonham MP, Taylor N, Duffy EM, Seamans K *et al*: **Estimation of the dietary requirement for vitamin D in free-living adults >=64 y of age**. *Am J Clin Nutr* 2009, **89**(5):1366-1374.
36. Cassady BA, Hollis JH, Fulford AD, Considine RV, Mattes RD: **Mastication of almonds: effects of lipid bioaccessibility, appetite, and hormone response**. *Am J Clin Nutr* 2009, **89**(3):794-800.
37. Castellanos VH, Marra MV, Johnson P: **Enhancement of select foods at breakfast and lunch increases energy intakes of nursing home residents with low meal intakes**. *J Am Diet Assoc* 2009, **109**(3):445-451.
38. Chaou CH, Chen CK, Chen JC, Chiu TF, Lin CC: **Comparisons of ice packs, hot water immersion, and analgesia injection for the treatment of centipede envenomations in Taiwan**. *Clin Toxicol (Phila)* 2009, **47**(7):659-662.
39. Chapman IM, Visvanathan R, Hammond AJ, Morley JE, Field JB, Tai K, Belobrajdic DP, Chen RY, Horowitz M: **Effect of testosterone and a nutritional supplement, alone and in combination, on hospital admissions in undernourished older men and women**. *Am J Clin Nutr* 2009, **89**(3):880-889.
40. Chase HP, Lutz K, Pencek R, Zhang B, Porter L: **Pramlintide lowered glucose excursions and was well-tolerated in adolescents with type 1 diabetes: results from a randomized, single-blind, placebo-controlled, crossover study**. *J Pediatr* 2009, **155**(3):369-373.
41. Cherkin DC, Sherman KJ, Avins AL, Erro JH, Ichikawa L, Barlow WE, Delaney K, Hawkes R, Hamilton L, Pressman A *et al*: **A randomized trial comparing acupuncture, simulated acupuncture, and usual care for chronic low back pain**. *Arch Intern Med* 2009, **169**(9):858-866.

42. Chermont AG, Falcao LF, de Souza Silva EH, de Cassia Xavier Balda R, Guinsburg R: **Skin-to-skin contact and/or oral 25% dextrose for procedural pain relief for term newborn infants**. *Pediatrics* 2009, **124**(6):e1101-1107.
43. Chevalier X, Goupille P, Beaulieu AD, Burch FX, Bensen WG, Conrozier T, Loeuille D, Kivitz AJ, Silver D, Appleton BE: **Intraarticular injection of anakinra in osteoarthritis of the knee: a multicenter, randomized, double-blind, placebo-controlled study**. *Arthritis Rheum* 2009, **61**(3):344-352.
44. Christian P, Shahid F, Rizvi A, Klemm RD, Bhutta ZA: **Treatment response to standard of care for severe anemia in pregnant women and effect of multivitamins and enhanced anthelminthics**. *Am J Clin Nutr* 2009, **89**(3):853-861.
45. Clark HD, Graham ID, Karovitch A, Keely EJ: **Do postal reminders increase postpartum screening of diabetes mellitus in women with gestational diabetes mellitus? A randomized controlled trial**. *Am J Obstet Gynecol* 2009, **200**(6):634 e631-637.
46. Cohen JA, Imrey PB, Calabresi PA, Edwards KR, Eickenhorst T, Felton WL, 3rd, Fisher E, Fox RJ, Goodman AD, Hara-Cleaver C *et al*: **Results of the Avonex Combination Trial (ACT) in relapsing-remitting MS**. *Neurology* 2009, **72**(6):535-541.
47. Cohen SB, Cheng TT, Chindalore V, Damjanov N, Burgos-Vargas R, Delora P, Zimany K, Travers H, Caulfield JP: **Evaluation of the efficacy and safety of pamapimod, a p38 MAP kinase inhibitor, in a double-blind, methotrexate-controlled study of patients with active rheumatoid arthritis**. *Arthritis Rheum* 2009, **60**(2):335-344.
48. Conklin HM, Lawford J, Jasper BW, Morris EB, Howard SC, Ogg SW, Wu S, Xiong X, Khan RB: **Side effects of methylphenidate in childhood cancer survivors: a randomized placebo-controlled trial**. *Pediatrics* 2009, **124**(1):226-233.
49. Connolly SJ, Ezekowitz MD, Yusuf S, Eikelboom J, Oldgren J, Parekh A, Pogue J, Reilly PA, Themeles E, Varrone J *et al*: **Dabigatran versus warfarin in patients with atrial fibrillation**. *N Engl J Med* 2009, **361**(12):1139-1151.
50. Connor KM, Shapiro RE, Diener HC, Lucas S, Kost J, Fan X, Fei K, Assaid C, Lines C, Ho TW: **Randomized, controlled trial of telcagepant for the acute treatment of migraine**. *Neurology* 2009, **73**(12):970-977.
51. Cordell WH, Maturi RK, Costigan TM, Marmor MF, Weleber RG, Coupland SG, Danis RP, McGettigan JW, Jr., Antoszyk AN, Klise S *et al*: **Retinal effects of 6 months of daily use of tadalafil or sildenafil**. *Arch Ophthalmol* 2009, **127**(4):367-373.
52. Cowling BJ, Chan KH, Fang VJ, Cheng CK, Fung RO, Wai W, Sin J, Seto WH, Yung R, Chu DW *et al*: **Facemasks and hand hygiene to prevent influenza transmission in households: a cluster randomized trial**. *Ann Intern Med* 2009, **151**(7):437-446.
53. Crawford MW, White MC, Propst EJ, Zaarour C, Cushing S, Pehora C, James AL, Gordon KA, Papsin BC: **Dose-dependent suppression of the electrically elicited stapedius reflex by general anesthetics in children undergoing cochlear implant surgery**. *Anesth Analg* 2009, **108**(5):1480-1487.
54. Cuvillon P, Nouvellon E, Ripart J, Boyer JC, Dehour L, Mahamat A, L'Hermite J, Boisson C, Vialles N, Lefrant JY *et al*: **A comparison of the pharmacodynamics and pharmacokinetics of bupivacaine, ropivacaine (with epinephrine) and their equal volume mixtures with lidocaine used for femoral and sciatic nerve blocks: a double-blind randomized study**. *Anesth Analg* 2009, **108**(2):641-649.

55. Dabu-Bondoc S, Vadivelu N, Benson J, Perret D, Kain ZN: **Hemispheric synchronized sounds and perioperative analgesic requirements**. *Anesth Analg*, **110**(1):208-210.
56. Dale KS, McAuley KA, Taylor RW, Williams SM, Farmer VL, Hansen P, Vorgers SM, Chisholm AW, Mann JI: **Determining optimal approaches for weight maintenance: a randomized controlled trial**. *Cmaj* 2009, **180**(10):E39-46.
57. Davidson LE, Hudson R, Kilpatrick K, Kuk JL, McMillan K, Janiszewski PM, Lee S, Lam M, Ross R: **Effects of exercise modality on insulin resistance and functional limitation in older adults: a randomized controlled trial**. *Arch Intern Med* 2009, **169**(2):122-131.
58. Davidsson L, Sarker SA, Jamil KA, Sultana S, Hurrell R: **Regular consumption of a complementary food fortified with ascorbic acid and ferrous fumarate or ferric pyrophosphate is as useful as ferrous sulfate in maintaining hemoglobin concentrations >105 g/L in young Bangladeshi children**. *Am J Clin Nutr* 2009, **89**(6):1815-1820.
59. Dawson-Hughes B, Harris SS, Palermo NJ, Castaneda-Sceppa C, Rasmussen HM, Dallal GE: **Treatment with potassium bicarbonate lowers calcium excretion and bone resorption in older men and women**. *J Clin Endocrinol Metab* 2009, **94**(1):96-102.
60. De Hert S, Vlasselaers D, Barbe R, Ory JP, Dekegel D, Donnadonni R, Demeere JL, Mulier J, Wouters P: **A comparison of volatile and non volatile agents for cardioprotection during on-pump coronary surgery**. *Anaesthesia* 2009, **64**(9):953-960.
61. de Smet AM, Kluytmans JA, Cooper BS, Mascini EM, Benus RF, van der Werf TS, van der Hoeven JG, Pickkers P, Bogaers-Hofman D, van der Meer NJ *et al*: **Decontamination of the digestive tract and oropharynx in ICU patients**. *N Engl J Med* 2009, **360**(1):20-31.
62. Dehghani SM, Erjaee A, Imanieh MH, Haghighat M: **Efficacy of the standard quadruple therapy versus triple therapies containing proton pump inhibitor plus amoxicillin and clarithromycin or amoxicillin-clavulanic acid and metronidazole for Helicobacter pylori eradication in children**. *Dig Dis Sci* 2009, **54**(8):1720-1724.
63. Dellinger RP, Tomayko JF, Angus DC, Opal S, Cupo MA, McDermott S, Ducher A, Calandra T, Cohen J: **Efficacy and safety of a phospholipid emulsion (GR270773) in Gram-negative severe sepsis: results of a phase II multicenter, randomized, placebo-controlled, dose-finding clinical trial**. *Crit Care Med* 2009, **37**(11):2929-2938.
64. Demmy TL, Nwogu C, Solan P, Yendamuri S, Wilding G, DeLeon O: **Chest tube-delivered bupivacaine improves pain and decreases opioid use after thoracoscopy**. *Ann Thorac Surg* 2009, **87**(4):1040-1046; discussion 1046-1047.
65. Dickinson JE, Doherty DA: **Optimization of third-stage management after second-trimester medical pregnancy termination**. *Am J Obstet Gynecol* 2009, **201**(3):303 e301-307.
66. Digenio AG, Mancuso JP, Gerber RA, Dvorak RV: **Comparison of methods for delivering a lifestyle modification program for obese patients: a randomized trial**. *Ann Intern Med* 2009, **150**(4):255-262.
67. Dimitriou VK, Zogogiannis ID, Douma AK, Pentilas ND, Liotiri DG, Wachtel MS, Karakitsos D: **Comparison of standard polyvinyl chloride tracheal tubes and straight reinforced tracheal tubes for tracheal intubation through different sizes of the Airtraq laryngoscope in anesthetized and paralyzed patients: a randomized prospective study**. *Anesthesiology* 2009, **111**(6):1265-1270.

68. Dromerick AW, Lang CE, Birkenmeier RL, Wagner JM, Miller JP, Videen TO, Powers WJ, Wolf SL, Edwards DF: **Very Early Constraint-Induced Movement during Stroke Rehabilitation (VECTORS): A single-center RCT**. *Neurology* 2009, **73**(3):195-201.
69. Dumont GJ, Kramers C, Sweep FC, Touw DJ, van Hasselt JG, de Kam M, van Gerven JM, Buitelaar JK, Verkes RJ: **Cannabis coadministration potentiates the effects of "ecstasy" on heart rate and temperature in humans**. *Clin Pharmacol Ther* 2009, **86**(2):160-166.
70. Dumville JC, Worthy G, Bland JM, Cullum N, Dowson C, Iglesias C, Mitchell JL, Nelson EA, Soares MO, Torgerson DJ: **Larval therapy for leg ulcers (VenUS II): randomised controlled trial**. *Bmj* 2009, **338**:b773.
71. Dunkelgrun M, Boersma E, Schouten O, Koopman-van Gemert AW, van Poorten F, Bax JJ, Thomson IR, Poldermans D: **Bisoprolol and fluvastatin for the reduction of perioperative cardiac mortality and myocardial infarction in intermediate-risk patients undergoing noncardiovascular surgery: a randomized controlled trial (DECREASE-IV)**. *Ann Surg* 2009, **249**(6):921-926.
72. Eberhardt F, Heringlake M, Massalme MS, Dyllus A, Misfeld M, Sievers HH, Wiegand UK, Hanke T: **The effect of biventricular pacing after coronary artery bypass grafting: a prospective randomized trial of different pacing modes in patients with reduced left ventricular function**. *J Thorac Cardiovasc Surg* 2009, **137**(6):1461-1467.
73. Ellerbeck EF, Mahnken JD, Cupertino AP, Cox LS, Greiner KA, Mussulman LM, Nazir N, Shireman TI, Resnicow K, Ahluwalia JS: **Effect of varying levels of disease management on smoking cessation: a randomized trial**. *Ann Intern Med* 2009, **150**(7):437-446.
74. Emery P, Fleischmann RM, Moreland LW, Hsia EC, Strusberg I, Durez P, Nash P, Amante EJ, Churchill M, Park W *et al*: **Golimumab, a human anti-tumor necrosis factor alpha monoclonal antibody, injected subcutaneously every four weeks in methotrexate-naive patients with active rheumatoid arthritis: twenty-four-week results of a phase III, multicenter, randomized, double-blind, placebo-controlled study of golimumab before methotrexate as first-line therapy for early-onset rheumatoid arthritis**. *Arthritis Rheum* 2009, **60**(8):2272-2283.
75. Fantin B, Duval X, Massias L, Alavoine L, Chau F, Retout S, Andremont A, Mentre F: **Ciprofloxacin dosage and emergence of resistance in human commensal bacteria**. *J Infect Dis* 2009, **200**(3):390-398.
76. Farkas D, Volak LP, Harmatz JS, von Moltke LL, Court MH, Greenblatt DJ: **Short-term clarithromycin administration impairs clearance and enhances pharmacodynamic effects of trazodone but not of zolpidem**. *Clin Pharmacol Ther* 2009, **85**(6):644-650.
77. Faucher JF, Aubouy A, Adeothy A, Cottrell G, Doritchamou J, Gourmel B, Houze P, Kossou H, Amedome H, Massougbodji A *et al*: **Comparison of sulfadoxine-pyrimethamine, unsupervised artemether-lumefantrine, and unsupervised artesunate-amodiaquine fixed-dose formulation for uncomplicated plasmodium falciparum malaria in Benin: a randomized effectiveness noninferiority trial**. *J Infect Dis* 2009, **200**(1):57-65.
78. Fernandez R, Compton S, Jones KA, Velilla MA: **The presence of a family witness impacts physician performance during simulated medical codes**. *Crit Care Med* 2009, **37**(6):1956-1960.

79. Ferrigno IS, Cliquet A, Jr., Magna LA, Zoppi Filho A: **Electromyography of the upper limbs during computer work: a comparison of 2 wrist orthoses in healthy adults**. *Arch Phys Med Rehabil* 2009, **90**(7):1152-1158.
80. Frassdorf J, Borowski A, Ebel D, Feindt P, Hermes M, Meemann T, Weber R, Mullenheim J, Weber NC, Preckel B *et al*: **Impact of preconditioning protocol on anesthetic-induced cardioprotection in patients having coronary artery bypass surgery**. *J Thorac Cardiovasc Surg* 2009, **137**(6):1436-1442, 1442 e1431-1432.
81. Freedland KE, Skala JA, Carney RM, Rubin EH, Lustman PJ, Davila-Roman VG, Steinmeyer BC, Hogue CW, Jr.: **Treatment of depression after coronary artery bypass surgery: a randomized controlled trial**. *Arch Gen Psychiatry* 2009, **66**(4):387-396.
82. Freer Y, McIntosh N, Teunisse S, Anand KJ, Boyle EM: **More information, less understanding: a randomized study on consent issues in neonatal research**. *Pediatrics* 2009, **123**(5):1301-1305.
83. Friedman BW, Bender B, Davitt M, Solorzano C, Paternoster J, Esses D, Bijur P, Gallagher EJ: **A randomized trial of diphenhydramine as prophylaxis against metoclopramide-induced akathisia in nauseated emergency department patients**. *Ann Emerg Med* 2009, **53**(3):379-385.
84. Frye RL, August P, Brooks MM, Hardison RM, Kelsey SF, MacGregor JM, Orchard TJ, Chaitman BR, Genuth SM, Goldberg SH *et al*: **A randomized trial of therapies for type 2 diabetes and coronary artery disease**. *N Engl J Med* 2009, **360**(24):2503-2515.
85. Fusar-Poli P, Crippa JA, Bhattacharyya S, Borgwardt SJ, Allen P, Martin-Santos R, Seal M, Surguladze SA, O'Carrol C, Atakan Z *et al*: **Distinct effects of {delta}9-tetrahydrocannabinol and cannabidiol on neural activation during emotional processing**. *Arch Gen Psychiatry* 2009, **66**(1):95-105.
86. Galie N, Brundage BH, Ghofrani HA, Oudiz RJ, Simonneau G, Safdar Z, Shapiro S, White RJ, Chan M, Beardsworth A *et al*: **Tadalafil therapy for pulmonary arterial hypertension**. *Circulation* 2009, **119**(22):2894-2903.
87. Garber A, Henry R, Ratner R, Garcia-Hernandez PA, Rodriguez-Pattzi H, Olvera-Alvarez I, Hale PM, Zdravkovic M, Bode B: **Liraglutide versus glimepiride monotherapy for type 2 diabetes (LEAD-3 Mono): a randomised, 52-week, phase III, double-blind, parallel-treatment trial**. *Lancet* 2009, **373**(9662):473-481.
88. Garra G, Singer AJ, Bamber D, Chohan J, Troxell R, Thode HC, Jr.: **Pretreatment of patients requiring oral contrast abdominal computed tomography with antiemetics: a randomized controlled trial of efficacy**. *Ann Emerg Med* 2009, **53**(4):528-533.
89. Gelotte CK, Prior MJ, Gu J: **A randomized, placebo-controlled, exploratory trial of Ibuprofen and pseudoephedrine in the treatment of primary nocturnal enuresis in children**. *Clin Pediatr (Phila)* 2009, **48**(4):410-419.
90. Giannarini G, Autorino R, Valent F, Mogorovich A, Manassero F, De Maria M, Morelli G, Barbone F, Di Lorenzo G, Selli C: **Combination of perianal-intrarectal lidocaine-prilocaine cream and periprostatic nerve block for pain control during transrectal ultrasound guided prostate biopsy: a randomized, controlled trial**. *J Urol* 2009, **181**(2):585-591; discussion 591-583.
91. Gilron I, Bailey JM, Tu D, Holden RR, Jackson AC, Houlden RL: **Nortriptyline and gabapentin, alone and in combination for neuropathic pain: a double-blind, randomised controlled crossover trial**. *Lancet* 2009, **374**(9697):1252-1261.
92. Gilron I, Orr E, Tu D, Mercer CD, Bond D: **A randomized, double-blind, controlled trial of perioperative administration of gabapentin, meloxicam and their**

combination for spontaneous and movement-evoked pain after ambulatory laparoscopic cholecystectomy. *Anesth Analg* 2009, **108**(2):623-630.
93. Ginzburg K, Butler LD, Giese-Davis J, Cavanaugh CE, Neri E, Koopman C, Classen CC, Spiegel D: **Shame, guilt, and posttraumatic stress disorder in adult survivors of childhood sexual abuse at risk for human immunodeficiency virus: outcomes of a randomized clinical trial of group psychotherapy treatment**. *J Nerv Ment Dis* 2009, **197**(7):536-542.
94. Gnant M, Mlineritsch B, Schippinger W, Luschin-Ebengreuth G, Postlberger S, Menzel C, Jakesz R, Seifert M, Hubalek M, Bjelic-Radisic V *et al*: **Endocrine therapy plus zoledronic acid in premenopausal breast cancer**. *N Engl J Med* 2009, **360**(7):679-691.
95. Goldberg AC, Bays HE, Ballantyne CM, Kelly MT, Buttler SM, Setze CM, Sleep DJ, Stolzenbach JC: **Efficacy and safety of ABT-335 (fenofibric acid) in combination with atorvastatin in patients with mixed dyslipidemia**. *Am J Cardiol* 2009, **103**(4):515-522.
96. Gormsen L, Finnerup NB, Almqvist PM, Jensen TS: **The efficacy of the AMPA receptor antagonist NS1209 and lidocaine in nerve injury pain: a randomized, double-blind, placebo-controlled, three-way crossover study**. *Anesth Analg* 2009, **108**(4):1311-1319.
97. Gosling RD, Gesase S, Mosha JF, Carneiro I, Hashim R, Lemnge M, Mosha FW, Greenwood B, Chandramohan D: **Protective efficacy and safety of three antimalarial regimens for intermittent preventive treatment for malaria in infants: a randomised, double-blind, placebo-controlled trial**. *Lancet* 2009, **374**(9700):1521-1532.
98. Gracies JM, Lugassy M, Weisz DJ, Vecchio M, Flanagan S, Simpson DM: **Botulinum toxin dilution and endplate targeting in spasticity: a double-blind controlled study**. *Arch Phys Med Rehabil* 2009, **90**(1):9-16 e12.
99. Gurbel PA, Bliden KP, Butler K, Tantry US, Gesheff T, Wei C, Teng R, Antonino MJ, Patil SB, Karunakaran A *et al*: **Randomized double-blind assessment of the ONSET and OFFSET of the antiplatelet effects of ticagrelor versus clopidogrel in patients with stable coronary artery disease: the ONSET/OFFSET study**. *Circulation* 2009, **120**(25):2577-2585.
100. Hahn CH, Rungby JA, Overgaard T, Moller H, Schultz P, Tos M: **Effect of diathermy on pain and healing in tonsillectomy, compared with other methods of haemostasis: a randomised study**. *J Laryngol Otol* 2009, **123**(6):648-655.
101. He L, Wang X, Zhang XF, Tang SR: **Effects of different doses of remifentanil on the end-tidal concentration of sevoflurane required for tracheal intubation in children**. *Anaesthesia* 2009, **64**(8):850-855.
102. Henry RR, Lincoff AM, Mudaliar S, Rabbia M, Chognot C, Herz M: **Effect of the dual peroxisome proliferator-activated receptor-alpha/gamma agonist aleglitazar on risk of cardiovascular disease in patients with type 2 diabetes (SYNCHRONY): a phase II, randomised, dose-ranging study**. *Lancet* 2009, **374**(9684):126-135.
103. Hezode C, Forestier N, Dusheiko G, Ferenci P, Pol S, Goeser T, Bronowicki JP, Bourliere M, Gharakhanian S, Bengtsson L *et al*: **Telaprevir and peginterferon with or without ribavirin for chronic HCV infection**. *N Engl J Med* 2009, **360**(18):1839-1850.
104. Hollander P, Li J, Allen E, Chen R: **Saxagliptin added to a thiazolidinedione improves glycemic control in patients with type 2 diabetes and inadequate**

control on thiazolidinedione alone. *J Clin Endocrinol Metab* 2009, **94**(12):4810-4819.
105. Hunt JR, Johnson LK, Fariba Roughead ZK: **Dietary protein and calcium interact to influence calcium retention: a controlled feeding study**. *Am J Clin Nutr* 2009, **89**(5):1357-1365.
106. Ibrahim SA, Hamido F, Al Misfer AK, Mahgoob A, Ghafar SA, Alhran H: **Anterior cruciate ligament reconstruction using autologous hamstring double bundle graft compared with single bundle procedures**. *J Bone Joint Surg Br* 2009, **91**(10):1310-1315.
107. Ip MS, Scott IU, VanVeldhuisen PC, Oden NL, Blodi BA, Fisher M, Singerman LJ, Tolentino M, Chan CK, Gonzalez VH: **A randomized trial comparing the efficacy and safety of intravitreal triamcinolone with observation to treat vision loss associated with macular edema secondary to central retinal vein occlusion: the Standard Care vs Corticosteroid for Retinal Vein Occlusion (SCORE) study report 5**. *Arch Ophthalmol* 2009, **127**(9):1101-1114.
108. Jaber S, Sebbane M, Verzilli D, Matecki S, Wysocki M, Eledjam JJ, Brochard L: **Adaptive support and pressure support ventilation behavior in response to increased ventilatory demand**. *Anesthesiology* 2009, **110**(3):620-627.
109. Jafar TH, Hatcher J, Poulter N, Islam M, Hashmi S, Qadri Z, Bux R, Khan A, Jafary FH, Hameed A *et al*: **Community-based interventions to promote blood pressure control in a developing country: a cluster randomized trial**. *Ann Intern Med* 2009, **151**(9):593-601.
110. Jan MH, Lin CH, Lin YF, Lin JJ, Lin DH: **Effects of weight-bearing versus nonweight-bearing exercise on function, walking speed, and position sense in participants with knee osteoarthritis: a randomized controlled trial**. *Arch Phys Med Rehabil* 2009, **90**(6):897-904.
111. Jenkinson CM, Doherty M, Avery AJ, Read A, Taylor MA, Sach TH, Silcocks P, Muir KR: **Effects of dietary intervention and quadriceps strengthening exercises on pain and function in overweight people with knee pain: randomised controlled trial**. *Bmj* 2009, **339**:b3170.
112. Jensen DM, Marcellin P, Freilich B, Andreone P, Di Bisceglie A, Brandao-Mello CE, Reddy KR, Craxi A, Martin AO, Teuber G *et al*: **Re-treatment of patients with chronic hepatitis C who do not respond to peginterferon-alpha2b: a randomized trial**. *Ann Intern Med* 2009, **150**(8):528-540.
113. Jeon WJ, Kim KH, Suh JK, Cho SY: **The use of remifentanil to facilitate the insertion of the Cobra perilaryngeal airway**. *Anesth Analg* 2009, **108**(5):1505-1509.
114. Johnston TE, Smith BT, Mulcahey MJ, Betz RR, Lauer RT: **A randomized controlled trial on the effects of cycling with and without electrical stimulation on cardiorespiratory and vascular health in children with spinal cord injury**. *Arch Phys Med Rehabil* 2009, **90**(8):1379-1388.
115. Jokela RM, Ahonen JV, Tallgren MK, Marjakangas PC, Korttila KT: **The effective analgesic dose of dexamethasone after laparoscopic hysterectomy**. *Anesth Analg* 2009, **109**(2):607-615.
116. Kaabachi O, Ouezini R, Koubaa W, Ghrab B, Zargouni A, Ben Abdelaziz A: **Tramadol as an adjuvant to lidocaine for axillary brachial plexus block**. *Anesth Analg* 2009, **108**(1):367-370.
117. Kain ZN, MacLaren JE, Herrmann L, Mayes L, Rosenbaum A, Hata J, Lerman J: **Preoperative melatonin and its effects on induction and emergence in children undergoing anesthesia and surgery**. *Anesthesiology* 2009, **111**(1):44-49.

118. Kavanaugh A, McInnes I, Mease P, Krueger GG, Gladman D, Gomez-Reino J, Papp K, Zrubek J, Mudivarthy S, Mack M *et al*: **Golimumab, a new human tumor necrosis factor alpha antibody, administered every four weeks as a subcutaneous injection in psoriatic arthritis: Twenty-four-week efficacy and safety results of a randomized, placebo-controlled study**. *Arthritis Rheum* 2009, **60**(4):976-986.
119. Kenny AM, Mangano KM, Abourizk RH, Bruno RS, Anamani DE, Kleppinger A, Walsh SJ, Prestwood KM, Kerstetter JE: **Soy proteins and isoflavones affect bone mineral density in older women: a randomized controlled trial**. *Am J Clin Nutr* 2009, **90**(1):234-242.
120. Kershaw TS, Magriples U, Westdahl C, Rising SS, Ickovics J: **Pregnancy as a window of opportunity for HIV prevention: effects of an HIV intervention delivered within prenatal care**. *Am J Public Health* 2009, **99**(11):2079-2086.
121. Khandanpour N, Armon MP, Jennings B, Finglas PM, Willis G, Clark A, Meyer FJ: **Randomized clinical trial of folate supplementation in patients with peripheral arterial disease**. *Br J Surg* 2009, **96**(9):990-998.
122. Khanna D, Clements PJ, Furst DE, Korn JH, Ellman M, Rothfield N, Wigley FM, Moreland LW, Silver R, Kim YH *et al*: **Recombinant human relaxin in the treatment of systemic sclerosis with diffuse cutaneous involvement: a randomized, double-blind, placebo-controlled trial**. *Arthritis Rheum* 2009, **60**(4):1102-1111.
123. Khazai NB, Judd SE, Jeng L, Wolfenden LL, Stecenko A, Ziegler TR, Tangpricha V: **Treatment and prevention of vitamin D insufficiency in cystic fibrosis patients: comparative efficacy of ergocalciferol, cholecalciferol, and UV light**. *J Clin Endocrinol Metab* 2009, **94**(6):2037-2043.
124. Kilic H, Zeytin HE, Korkmaz C, Mat C, Gul A, Cosan F, Dinc A, Simsek I, Sut N, Yazici H: **Low-dose natural human interferon-alpha lozenges in the treatment of Behcet's syndrome**. *Rheumatology (Oxford)* 2009, **48**(11):1388-1391.
125. Kim JW, Seo HS, Park JH, Na JO, Choi CU, Lim HE, Kim EJ, Rha SW, Park CG, Oh DJ: **A prospective, randomized, 6-month comparison of the coronary vasomotor response associated with a zotarolimus- versus a sirolimus-eluting stent: differential recovery of coronary endothelial dysfunction**. *J Am Coll Cardiol* 2009, **53**(18):1653-1659.
126. Kim KS, Kim KN, Hwang KG, Park CJ: **Capsicum plaster at the Hegu point reduces postoperative analgesic requirement after orthognathic surgery**. *Anesth Analg* 2009, **108**(3):992-996.
127. Kitzman-Ulrich H, Hampson R, Wilson DK, Presnell K, Brown A, O'Boyle M: **An adolescent weight-loss program integrating family variables reduces energy intake**. *J Am Diet Assoc* 2009, **109**(3):491-496.
128. Ko R, McRae K, Darling G, Waddell TK, McGlade D, Cheung K, Katz J, Slinger P: **The use of air in the inspired gas mixture during two-lung ventilation delays lung collapse during one-lung ventilation**. *Anesth Analg* 2009, **108**(4):1092-1096.
129. Kocsis JH, Gelenberg AJ, Rothbaum BO, Klein DN, Trivedi MH, Manber R, Keller MB, Leon AC, Wisniewski SR, Arnow BA *et al*: **Cognitive behavioral analysis system of psychotherapy and brief supportive psychotherapy for augmentation of antidepressant nonresponse in chronic depression: the REVAMP Trial**. *Arch Gen Psychiatry* 2009, **66**(11):1178-1188.
130. Komoroski B, Vachharajani N, Feng Y, Li L, Kornhauser D, Pfister M: **Dapagliflozin, a novel, selective SGLT2 inhibitor, improved glycemic control over 2 weeks in patients with type 2 diabetes mellitus**. *Clin Pharmacol Ther* 2009, **85**(5):513-519.

131. Kremer JM, Bloom BJ, Breedveld FC, Coombs JH, Fletcher MP, Gruben D, Krishnaswami S, Burgos-Vargas R, Wilkinson B, Zerbini CA *et al*: **The safety and efficacy of a JAK inhibitor in patients with active rheumatoid arthritis: Results of a double-blind, placebo-controlled phase IIa trial of three dosage levels of CP-690,550 versus placebo**. *Arthritis Rheum* 2009, **60**(7):1895-1905.
132. Kuijper B, Tans JT, Beelen A, Nollet F, de Visser M: **Cervical collar or physiotherapy versus wait and see policy for recent onset cervical radiculopathy: randomised trial**. *Bmj* 2009, **339**:b3883.
133. Kulig K, Reischl SF, Pomrantz AB, Burnfield JM, Mais-Requejo S, Thordarson DB, Smith RW: **Nonsurgical management of posterior tibial tendon dysfunction with orthoses and resistive exercise: a randomized controlled trial**. *Phys Ther* 2009, **89**(1):26-37.
134. La Gamma EF, van Wassenaer AG, Ares S, Golombek SG, Kok JH, Quero J, Hong T, Rahbar MH, de Escobar GM, Fisher DA *et al*: **Phase 1 trial of 4 thyroid hormone regimens for transient hypothyroxinemia in neonates of <28 weeks' gestation**. *Pediatrics* 2009, **124**(2):e258-268.
135. Landberg R, Aman P, Friberg LE, Vessby B, Adlercreutz H, Kamal-Eldin A: **Dose response of whole-grain biomarkers: alkylresorcinols in human plasma and their metabolites in urine in relation to intake**. *Am J Clin Nutr* 2009, **89**(1):290-296.
136. Lapperre TS, Snoeck-Stroband JB, Gosman MM, Jansen DF, van Schadewijk A, Thiadens HA, Vonk JM, Boezen HM, Ten Hacken NH, Sont JK *et al*: **Effect of fluticasone with and without salmeterol on pulmonary outcomes in chronic obstructive pulmonary disease: a randomized trial**. *Ann Intern Med* 2009, **151**(8):517-527.
137. Laughon M, Bose C, Moya F, Aschner J, Donn SM, Morabito C, Cummings JJ, Segal R, Guardia C, Liu G: **A pilot randomized, controlled trial of later treatment with a peptide-containing, synthetic surfactant for the prevention of bronchopulmonary dysplasia**. *Pediatrics* 2009, **123**(1):89-96.
138. Lee CW, Park DW, Lee SH, Kim YH, Hong MK, Kim JJ, Park SW, Yun SC, Seong IW, Lee JH *et al*: **Comparison of the efficacy and safety of zotarolimus-, sirolimus-, and paclitaxel-eluting stents in patients with ST-elevation myocardial infarction**. *Am J Cardiol* 2009, **104**(10):1370-1376.
139. Lee YY, Ngan Kee WD, Fong SY, Liu JT, Gin T: **The median effective dose of bupivacaine, levobupivacaine, and ropivacaine after intrathecal injection in lower limb surgery**. *Anesth Analg* 2009, **109**(4):1331-1334.
140. Lenhardt R, Orhan-Sungur M, Komatsu R, Govinda R, Kasuya Y, Sessler DI, Wadhwa A: **Suppression of shivering during hypothermia using a novel drug combination in healthy volunteers**. *Anesthesiology* 2009, **111**(1):110-115.
141. Leo S, Sng BL, Lim Y, Sia AT: **A randomized comparison of low doses of hyperbaric bupivacaine in combined spinal-epidural anesthesia for cesarean delivery**. *Anesth Analg* 2009, **109**(5):1600-1605.
142. Lewis D, Winner P, Saper J, Ness S, Polverejan E, Wang S, Kurland CL, Nye J, Yuen E, Eerdekens M *et al*: **Randomized, double-blind, placebo-controlled study to evaluate the efficacy and safety of topiramate for migraine prevention in pediatric subjects 12 to 17 years of age**. *Pediatrics* 2009, **123**(3):924-934.
143. Leyer GJ, Li S, Mubasher ME, Reifer C, Ouwehand AC: **Probiotic effects on cold and influenza-like symptom incidence and duration in children**. *Pediatrics* 2009, **124**(2):e172-179.
144. Lindeman JH, Abdul-Hussien H, van Bockel JH, Wolterbeek R, Kleemann R: **Clinical trial of doxycycline for matrix metalloproteinase-9 inhibition in patients with an**

abdominal aneurysm: doxycycline selectively depletes aortic wall neutrophils and cytotoxic T cells. *Circulation* 2009, **119**(16):2209-2216.
145. Ling BS, Schoen RE, Trauth JM, Wahed AS, Eury T, Simak DM, Solano FX, Weissfeld JL: **Physicians encouraging colorectal screening: a randomized controlled trial of enhanced office and patient management on compliance with colorectal cancer screening**. *Arch Intern Med* 2009, **169**(1):47-55.
146. Lingeman JE, Preminger GM, Goldfischer ER, Krambeck AE: **Assessing the impact of ureteral stent design on patient comfort**. *J Urol* 2009, **181**(6):2581-2587.
147. Liu PY, Liu YW, Lin LJ, Chen JH, Liao JK: **Evidence for statin pleiotropy in humans: differential effects of statins and ezetimibe on rho-associated coiled-coil containing protein kinase activity, endothelial function, and inflammation**. *Circulation* 2009, **119**(1):131-138.
148. Lonn EM, Gerstein HC, Sheridan P, Smith S, Diaz R, Mohan V, Bosch J, Yusuf S, Dagenais GR: **Effect of ramipril and of rosiglitazone on carotid intima-media thickness in people with impaired glucose tolerance or impaired fasting glucose: STARR (STudy of Atherosclerosis with Ramipril and Rosiglitazone)**. *J Am Coll Cardiol* 2009, **53**(22):2028-2035.
149. Lu LJ, Bao CD, Dai M, Teng JL, Fan W, Du F, Yang NP, Zhao YH, Chen ZW, Xu JH *et al*: **Multicenter, randomized, double-blind, controlled trial of treatment of active rheumatoid arthritis with T-614 compared with methotrexate**. *Arthritis Rheum* 2009, **61**(7):979-987.
150. Maassen R, Lee R, Hermans B, Marcus M, van Zundert A: **A comparison of three videolaryngoscopes: the Macintosh laryngoscope blade reduces, but does not replace, routine stylet use for intubation in morbidly obese patients**. *Anesth Analg* 2009, **109**(5):1560-1565.
151. Mahabadi V, Amory JK, Swerdloff RS, Bremner WJ, Page ST, Sitruk-Ware R, Christensen PD, Kumar N, Tsong YY, Blithe D *et al*: **Combined transdermal testosterone gel and the progestin nestorone suppresses serum gonadotropins in men**. *J Clin Endocrinol Metab* 2009, **94**(7):2313-2320.
152. Malik JA, Gupta D, Agarwal AN, Jindal SK: **Anticholinergic premedication for flexible bronchoscopy: a randomized, double-blind, placebo-controlled study of atropine and glycopyrrolate**. *Chest* 2009, **136**(2):347-354.
153. Malik P, Balaban DH, Thompson WO, Galt DJ: **Randomized study comparing two regimens of oral sodium phosphates solution versus low-dose polyethylene glycol and bisacodyl**. *Dig Dis Sci* 2009, **54**(4):833-841.
154. Mand S, Pfarr K, Sahoo PK, Satapathy AK, Specht S, Klarmann U, Debrah AY, Ravindran B, Hoerauf A: **Macrofilaricidal activity and amelioration of lymphatic pathology in bancroftian filariasis after 3 weeks of doxycycline followed by single-dose diethylcarbamazine**. *Am J Trop Med Hyg* 2009, **81**(4):702-711.
155. Manzoni GM, Pagnini F, Gorini A, Preziosa A, Castelnuovo G, Molinari E, Riva G: **Can relaxation training reduce emotional eating in women with obesity? An exploratory study with 3 months of follow-up**. *J Am Diet Assoc* 2009, **109**(8):1427-1432.
156. Manzoni P, Rinaldi M, Cattani S, Pugni L, Romeo MG, Messner H, Stolfi I, Decembrino L, Laforgia N, Vagnarelli F *et al*: **Bovine lactoferrin supplementation for prevention of late-onset sepsis in very low-birth-weight neonates: a randomized trial**. *Jama* 2009, **302**(13):1421-1428.
157. Marbury TC, Jin B, Panebianco D, Murphy MG, Sun H, Evans JK, Han TH, Constanzer ML, Dru J, Shadle CR: **Lack of effect of aprepitant or its prodrug**

fosaprepitant on QTc intervals in healthy subjects. *Anesth Analg* 2009, **109**(2):418-425.

158. Marino J, Russo J, Kenny M, Herenstein R, Livote E, Chelly JE: **Continuous lumbar plexus block for postoperative pain control after total hip arthroplasty. A randomized controlled trial**. *J Bone Joint Surg Am* 2009, **91**(1):29-37.
159. Massie BM, Collins JF, Ammon SE, Armstrong PW, Cleland JG, Ezekowitz M, Jafri SM, Krol WF, O'Connor CM, Schulman KA *et al*: **Randomized trial of warfarin, aspirin, and clopidogrel in patients with chronic heart failure: the Warfarin and Antiplatelet Therapy in Chronic Heart Failure (WATCH) trial**. *Circulation* 2009, **119**(12):1616-1624.
160. Masui K, Kira M, Kazama T, Hagihira S, Mortier EP, Struys MM: **Early phase pharmacokinetics but not pharmacodynamics are influenced by propofol infusion rate**. *Anesthesiology* 2009, **111**(4):805-817.
161. Mathias AA, West S, Hui J, Kearney BP: **Dose-response of ritonavir on hepatic CYP3A activity and elvitegravir oral exposure**. *Clin Pharmacol Ther* 2009, **85**(1):64-70.
162. Matoba M, Kitadate M, Kondou T, Yokota H, Tonami H: **Depiction of hypervascular hepatocellular carcinoma with 64-MDCT: comparison of moderate- and high-concentration contrast material with and without saline flush**. *AJR Am J Roentgenol* 2009, **193**(3):738-744.
163. Mattsson C, Reynolds RM, Simonyte K, Olsson T, Walker BR: **Combined receptor antagonist stimulation of the hypothalamic-pituitary-adrenal axis test identifies impaired negative feedback sensitivity to cortisol in obese men**. *J Clin Endocrinol Metab* 2009, **94**(4):1347-1352.
164. Mauer M, Zinman B, Gardiner R, Suissa S, Sinaiko A, Strand T, Drummond K, Donnelly S, Goodyer P, Gubler MC *et al*: **Renal and retinal effects of enalapril and losartan in type 1 diabetes**. *N Engl J Med* 2009, **361**(1):40-51.
165. McAlister FA, Fradette M, Majumdar SR, Williams R, Graham M, McMeekin J, Ghali WA, Tsuyuki RT, Knudtson ML, Grimshaw J: **The Enhancing Secondary Prevention in Coronary Artery Disease trial**. *Cmaj* 2009, **181**(12):897-904.
166. McCall DO, McGartland CP, McKinley MC, Patterson CC, Sharpe P, McCance DR, Young IS, Woodside JV: **Dietary intake of fruits and vegetables improves microvascular function in hypertensive subjects in a dose-dependent manner**. *Circulation* 2009, **119**(16):2153-2160.
167. McDermott MM, Ades P, Guralnik JM, Dyer A, Ferrucci L, Liu K, Nelson M, Lloyd-Jones D, Van Horn L, Garside D *et al*: **Treadmill exercise and resistance training in patients with peripheral arterial disease with and without intermittent claudication: a randomized controlled trial**. *Jama* 2009, **301**(2):165-174.
168. McHutchison JG, Everson GT, Gordon SC, Jacobson IM, Sulkowski M, Kauffman R, McNair L, Alam J, Muir AJ: **Telaprevir with peginterferon and ribavirin for chronic HCV genotype 1 infection**. *N Engl J Med* 2009, **360**(18):1827-1838.
169. McHutchison JG, Lawitz EJ, Shiffman ML, Muir AJ, Galler GW, McCone J, Nyberg LM, Lee WM, Ghalib RH, Schiff ER *et al*: **Peginterferon alfa-2b or alfa-2a with ribavirin for treatment of hepatitis C infection**. *N Engl J Med* 2009, **361**(6):580-593.
170. McIntosh MS, Konzelmann J, Smith J, Kalynych CJ, Wears RL, Schneider H, Wylie T, Kaminski A, Matar-Joseph M: **Stabilization and treatment of dental avulsions and fractures by emergency physicians using just-in-time training**. *Ann Emerg Med* 2009, **54**(4):585-592.

171. McNaughton C, Zhou C, Robert L, Storrow A, Kennedy R: **A randomized, crossover comparison of injected buffered lidocaine, lidocaine cream, and no analgesia for peripheral intravenous cannula insertion**. *Ann Emerg Med* 2009, **54**(2):214-220.
172. Meneghini RM, Smits SA: **Early discharge and recovery with three minimally invasive total hip arthroplasty approaches: a preliminary study**. *Clin Orthop Relat Res* 2009, **467**(6):1431-1437.
173. Menza M, Dobkin RD, Marin H, Mark MH, Gara M, Buyske S, Bienfait K, Dicke A: **A controlled trial of antidepressants in patients with Parkinson disease and depression**. *Neurology* 2009, **72**(10):886-892.
174. Meyhoff CS, Lund J, Jenstrup MT, Claudius C, Sorensen AM, Viby-Mogensen J, Rasmussen LS: **Should dosing of rocuronium in obese patients be based on ideal or corrected body weight?** *Anesth Analg* 2009, **109**(3):787-792.
175. Milgrom P, Ly KA, Tut OK, Mancl L, Roberts MC, Briand K, Gancio MJ: **Xylitol pediatric topical oral syrup to prevent dental caries: a double-blind randomized clinical trial of efficacy**. *Arch Pediatr Adolesc Med* 2009, **163**(7):601-607.
176. Miller M, Beach V, Sorkin JD, Mangano C, Dobmeier C, Novacic D, Rhyne J, Vogel RA: **Comparative effects of three popular diets on lipids, endothelial function, and C-reactive protein during weight maintenance**. *J Am Diet Assoc* 2009, **109**(4):713-717.
177. Moerman AT, Herregods LL, De Vos MM, Mortier EP, Struys MM: **Manual versus target-controlled infusion remifentanil administration in spontaneously breathing patients**. *Anesth Analg* 2009, **108**(3):828-834.
178. Mohiuddin SM, Pepine CJ, Kelly MT, Buttler SM, Setze CM, Sleep DJ, Stolzenbach JC: **Efficacy and safety of ABT-335 (fenofibric acid) in combination with simvastatin in patients with mixed dyslipidemia: a phase 3, randomized, controlled study**. *Am Heart J* 2009, **157**(1):195-203.
179. Mohta M, Kumari N, Tyagi A, Sethi AK, Agarwal D, Singh M: **Tramadol for prevention of postanaesthetic shivering: a randomised double-blind comparison with pethidine**. *Anaesthesia* 2009, **64**(2):141-146.
180. Movafegh A, Nouralishahi B, Sadeghi M, Nabavian O: **An ultra-low dose of naloxone added to lidocaine or lidocaine-fentanyl mixture prolongs axillary brachial plexus blockade**. *Anesth Analg* 2009, **109**(5):1679-1683.
181. Muthayya S, Eilander A, Transler C, Thomas T, van der Knaap HC, Srinivasan K, van Klinken BJ, Osendarp SJ, Kurpad AV: **Effect of fortification with multiple micronutrients and n-3 fatty acids on growth and cognitive performance in Indian schoolchildren: the CHAMPION (Children's Health and Mental Performance Influenced by Optimal Nutrition) Study**. *Am J Clin Nutr* 2009, **89**(6):1766-1775.
182. Narayanaswamy M, McRae K, Slinger P, Dugas G, Kanellakos GW, Roscoe A, Lacroix M: **Choosing a lung isolation device for thoracic surgery: a randomized trial of three bronchial blockers versus double-lumen tubes**. *Anesth Analg* 2009, **108**(4):1097-1101.
183. Nasso G, Coppola R, Bonifazi R, Piancone F, Bozzetti G, Speziale G: **Arterial revascularization in primary coronary artery bypass grafting: Direct comparison of 4 strategies--results of the Stand-in-Y Mammary Study**. *J Thorac Cardiovasc Surg* 2009, **137**(5):1093-1100.
184. Nicklas BJ, Wang X, You T, Lyles MF, Demons J, Easter L, Berry MJ, Lenchik L, Carr JJ: **Effect of exercise intensity on abdominal fat loss during calorie restriction in overweight and obese postmenopausal women: a randomized, controlled trial**. *Am J Clin Nutr* 2009, **89**(4):1043-1052.

185. Noehr-Jensen L, Zwisler ST, Larsen F, Sindrup SH, Damkier P, Brosen K: **Escitalopram is a weak inhibitor of the CYP2D6-catalyzed O-demethylation of (+)-tramadol but does not reduce the hypoalgesic effect in experimental pain**. *Clin Pharmacol Ther* 2009, **86**(6):626-633.
186. Novak I, Cusick A, Lannin N: **Occupational therapy home programs for cerebral palsy: double-blind, randomized, controlled trial**. *Pediatrics* 2009, **124**(4):e606-614.
187. Nucifora G, Badano LP, Sarraf-Zadegan N, Karavidas A, Trocino G, Scaffidi G, Pettinati G, Astarita C, Vysniauskas V, Gregori D *et al*: **Effect on quality of life of different accelerated diagnostic protocols for management of patients presenting to the emergency department with acute chest pain**. *Am J Cardiol* 2009, **103**(5):592-597.
188. Nyamathi A, Liu Y, Marfisee M, Shoptaw S, Gregerson P, Saab S, Leake B, Tyler D, Gelberg L: **Effects of a nurse-managed program on hepatitis A and B vaccine completion among homeless adults**. *Nurs Res* 2009, **58**(1):13-22.
189. Nyunt MM, Hendrix CW, Bakshi RP, Kumar N, Shapiro TA: **Phase I/II evaluation of the prophylactic antimalarial activity of pafuramidine in healthy volunteers challenged with Plasmodium falciparum sporozoites**. *Am J Trop Med Hyg* 2009, **80**(4):528-535.
190. O'Byrne PM, van der Linde J, Cockcroft DW, Gauvreau GM, Brannan JD, Fitzgerald M, Watson RM, Milot J, Davis B, O'Connor M *et al*: **Prolonged bronchoprotection against inhaled methacholine by inhaled BI 1744, a long-acting beta(2)-agonist, in patients with mild asthma**. *J Allergy Clin Immunol* 2009, **124**(6):1217-1221.
191. Ohrvik VE, Olsson JC, Sundberg BE, Witthoft CM: **Effect of 2 pieces of nutritional advice on folate status in Swedish women: a randomized controlled trial**. *Am J Clin Nutr* 2009, **89**(4):1053-1058.
192. Olanow CW, Rascol O, Hauser R, Feigin PD, Jankovic J, Lang A, Langston W, Melamed E, Poewe W, Stocchi F *et al*: **A double-blind, delayed-start trial of rasagiline in Parkinson's disease**. *N Engl J Med* 2009, **361**(13):1268-1278.
193. Olson LC, Hong D, Conell-Price JS, Cheng S, Flood P: **A transdermal nicotine patch is not effective for postoperative pain management in smokers: a pilot dose-ranging study**. *Anesth Analg* 2009, **109**(6):1987-1991.
194. Otsuki M, Eakin MN, Rand CS, Butz AM, Hsu VD, Zuckerman IH, Ogborn J, Bilderback A, Riekert KA: **Adherence feedback to improve asthma outcomes among inner-city children: a randomized trial**. *Pediatrics* 2009, **124**(6):1513-1521.
195. Oyer DS, Shepherd MD, Coulter FC, Bhargava A, Brett J, Chu PL, Trippe BS: **A(1c) control in a primary care setting: self-titrating an insulin analog pre-mix (INITIATEplus trial)**. *Am J Med* 2009, **122**(11):1043-1049.
196. Panousis P, Heller AR, Koch T, Litz RJ: **Epidural ropivacaine concentrations for intraoperative analgesia during major upper abdominal surgery: a prospective, randomized, double-blinded, placebo-controlled study**. *Anesth Analg* 2009, **108**(6):1971-1976.
197. Pantalitschka T, Sievers J, Urschitz MS, Herberts T, Reher C, Poets CF: **Randomised crossover trial of four nasal respiratory support systems for apnoea of prematurity in very low birthweight infants**. *Arch Dis Child Fetal Neonatal Ed* 2009, **94**(4):F245-248.
198. Park SH, Han SH, Do SH, Kim JW, Kim JH: **The influence of head and neck position on the oropharyngeal leak pressure and cuff position of three supraglottic airway devices**. *Anesth Analg* 2009, **108**(1):112-117.

199. Paterna S, Parrinello G, Cannizzaro S, Fasullo S, Torres D, Sarullo FM, Di Pasquale P: **Medium term effects of different dosage of diuretic, sodium, and fluid administration on neurohormonal and clinical outcome in patients with recently compensated heart failure**. *Am J Cardiol* 2009, **103**(1):93-102.
200. Pempek TA, Calvert SL: **Tipping the balance: use of advergames to promote consumption of nutritious foods and beverages by low-income African American children**. *Arch Pediatr Adolesc Med* 2009, **163**(7):633-637.
201. Peters HP, Boers HM, Haddeman E, Melnikov SM, Qvyjt F: **No effect of added beta-glucan or of fructooligosaccharide on appetite or energy intake**. *Am J Clin Nutr* 2009, **89**(1):58-63.
202. Peterson CB, Mitchell JE, Crow SJ, Crosby RD, Wonderlich SA: **The efficacy of self-help group treatment and therapist-led group treatment for binge eating disorder**. *Am J Psychiatry* 2009, **166**(12):1347-1354.
203. Piper ME, Smith SS, Schlam TR, Fiore MC, Jorenby DE, Fraser D, Baker TB: **A randomized placebo-controlled clinical trial of 5 smoking cessation pharmacotherapies**. *Arch Gen Psychiatry* 2009, **66**(11):1253-1262.
204. Pisegna JR, Karlstadt RG, Norton JA, Fogel R, Oh DS, Jay Graepel G, Dorr MB: **Effect of preoperative intravenous pantoprazole in elective-surgery patients: a pilot study**. *Dig Dis Sci* 2009, **54**(5):1041-1049.
205. Plaud B, Meretoja O, Hofmockel R, Raft J, Stoddart PA, van Kuijk JH, Hermens Y, Mirakhur RK: **Reversal of rocuronium-induced neuromuscular blockade with sugammadex in pediatric and adult surgical patients**. *Anesthesiology* 2009, **110**(2):284-294.
206. Plint AC, Johnson DW, Patel H, Wiebe N, Correll R, Brant R, Mitton C, Gouin S, Bhatt M, Joubert G *et al*: **Epinephrine and dexamethasone in children with bronchiolitis**. *N Engl J Med* 2009, **360**(20):2079-2089.
207. Pot GK, Majsak-Newman G, Geelen A, Harvey LJ, Nagengast FM, Witteman BJ, van de Meeberg PC, Timmer R, Tan A, Wahab PJ *et al*: **Fish consumption and markers of colorectal cancer risk: a multicenter randomized controlled trial**. *Am J Clin Nutr* 2009, **90**(2):354-361.
208. Pradhan AD, Everett BM, Cook NR, Rifai N, Ridker PM: **Effects of initiating insulin and metformin on glycemic control and inflammatory biomarkers among patients with type 2 diabetes: the LANCET randomized trial**. *Jama* 2009, **302**(11):1186-1194.
209. Puhl R, Wharton C, Heuer C: **Weight bias among dietetics students: implications for treatment practices**. *J Am Diet Assoc* 2009, **109**(3):438-444.
210. Rahmann AE, Brauer SG, Nitz JC: **A specific inpatient aquatic physiotherapy program improves strength after total hip or knee replacement surgery: a randomized controlled trial**. *Arch Phys Med Rehabil* 2009, **90**(5):745-755.
211. Rajaram S, Haddad EH, Mejia A, Sabate J: **Walnuts and fatty fish influence different serum lipid fractions in normal to mildly hyperlipidemic individuals: a randomized controlled study**. *Am J Clin Nutr* 2009, **89**(5):1657S-1663S.
212. Reinius H, Jonsson L, Gustafsson S, Sundbom M, Duvernoy O, Pelosi P, Hedenstierna G, Freden F: **Prevention of atelectasis in morbidly obese patients during general anesthesia and paralysis: a computerized tomography study**. *Anesthesiology* 2009, **111**(5):979-987.
213. Richter S, Kollmar O, Schuld J, Moussavian MR, Igna D, Schilling MK: **Randomized clinical trial of efficacy and costs of three dissection devices in liver resection**. *Br J Surg* 2009, **96**(6):593-601.

214. Riesmeier A, Schellhaass A, Boldt J, Suttner S: **Crystalloid/colloid versus crystalloid intravascular volume administration before spinal anesthesia in elderly patients: the influence on cardiac output and stroke volume**. *Anesth Analg* 2009, **108**(2):650-654.
215. Rimmer JH, Rauworth AE, Wang EC, Nicola TL, Hill B: **A preliminary study to examine the effects of aerobic and therapeutic (nonaerobic) exercise on cardiorespiratory fitness and coronary risk reduction in stroke survivors**. *Arch Phys Med Rehabil* 2009, **90**(3):407-412.
216. Roh JL, Park JY, Park CI: **Prevention of postoperative hypocalcemia with routine oral calcium and vitamin D supplements in patients with differentiated papillary thyroid carcinoma undergoing total thyroidectomy plus central neck dissection**. *Cancer* 2009, **115**(2):251-258.
217. Rosenson RS, Hislop C, McConnell D, Elliott M, Stasiv Y, Wang N, Waters DD: **Effects of 1-H-indole-3-glyoxamide (A-002) on concentration of secretory phospholipase A2 (PLASMA study): a phase II double-blind, randomised, placebo-controlled trial**. *Lancet* 2009, **373**(9664):649-658.
218. Rossignol JF, Elfert A, El-Gohary Y, Keeffe EB: **Improved virologic response in chronic hepatitis C genotype 4 treated with nitazoxanide, peginterferon, and ribavirin**. *Gastroenterology* 2009, **136**(3):856-862.
219. Rossini M, Viapiana O, Ramonda R, Bianchi G, Olivieri I, Lapadula G, Adami S: **Intra-articular clodronate for the treatment of knee osteoarthritis: dose ranging study vs hyaluronic acid**. *Rheumatology (Oxford)* 2009, **48**(7):773-778.
220. Rusch LC, Kanter JW, Brondino MJ: **A comparison of contextual and biomedical models of stigma reduction for depression with a nonclinical undergraduate sample**. *J Nerv Ment Dis* 2009, **197**(2):104-110.
221. Russell IJ, Perkins AT, Michalek JE: **Sodium oxybate relieves pain and improves function in fibromyalgia syndrome: a randomized, double-blind, placebo-controlled, multicenter clinical trial**. *Arthritis Rheum* 2009, **60**(1):299-309.
222. Sabatine MS, Antman EM, Widimsky P, Ebrahim IO, Kiss RG, Saaiman A, Polasek R, Contant CF, McCabe CH, Braunwald E: **Otamixaban for the treatment of patients with non-ST-elevation acute coronary syndromes (SEPIA-ACS1 TIMI 42): a randomised, double-blind, active-controlled, phase 2 trial**. *Lancet* 2009, **374**(9692):787-795.
223. Sackeim HA, Dillingham EM, Prudic J, Cooper T, McCall WV, Rosenquist P, Isenberg K, Garcia K, Mulsant BH, Haskett RF: **Effect of concomitant pharmacotherapy on electroconvulsive therapy outcomes: short-term efficacy and adverse effects**. *Arch Gen Psychiatry* 2009, **66**(7):729-737.
224. Sacks FM, Bray GA, Carey VJ, Smith SR, Ryan DH, Anton SD, McManus K, Champagne CM, Bishop LM, Laranjo N *et al*: **Comparison of weight-loss diets with different compositions of fat, protein, and carbohydrates**. *N Engl J Med* 2009, **360**(9):859-873.
225. Safarinejad MR, Safarinejad S: **Efficacy of selenium and/or N-acetyl-cysteine for improving semen parameters in infertile men: a double-blind, placebo controlled, randomized study**. *J Urol* 2009, **181**(2):741-751.
226. Saltzman MD, Nuber GW, Gryzlo SM, Marecek GS, Koh JL: **Efficacy of surgical preparation solutions in shoulder surgery**. *J Bone Joint Surg Am* 2009, **91**(8):1949-1953.
227. Santilli F, Rocca B, De Cristofaro R, Lattanzio S, Pietrangelo L, Habib A, Pettinella C, Recchiuti A, Ferrante E, Ciabattoni G *et al*: **Platelet cyclooxygenase inhibition by**

low-dose aspirin is not reflected consistently by platelet function assays: implications for aspirin "resistance". *J Am Coll Cardiol* 2009, **53**(8):667-677.

228. Sattler FR, Castaneda-Sceppa C, Binder EF, Schroeder ET, Wang Y, Bhasin S, Kawakubo M, Stewart Y, Yarasheski KE, Ulloor J *et al*: **Testosterone and growth hormone improve body composition and muscle performance in older men**. *J Clin Endocrinol Metab* 2009, **94**(6):1991-2001.
229. Sayegh FE, Kenanidis EI, Papavasiliou KA, Potoupnis ME, Kirkos JM, Kapetanos GA: **Reduction of acute anterior dislocations: a prospective randomized study comparing a new technique with the Hippocratic and Kocher methods**. *J Bone Joint Surg Am* 2009, **91**(12):2775-2782.
230. Scalabrin DM, Johnston WH, Hoffman DR, P'Pool VL, Harris CL, Mitmesser SH: **Growth and tolerance of healthy term infants receiving hydrolyzed infant formulas supplemented with Lactobacillus rhamnosus GG: randomized, double-blind, controlled trial**. *Clin Pediatr (Phila)* 2009, **48**(7):734-744.
231. Scheller BC, Daunderer M, Pipa G: **General anesthesia increases temporal precision and decreases power of the brainstem auditory-evoked response-related segments of the electroencephalogram**. *Anesthesiology* 2009, **111**(2):340-355.
232. Schmidl D, Polska E, Kiss B, Sacu S, Garhofer G, Schmetterer L: **Ocular hemodynamic effects of nitrovasodilators in healthy subjects**. *Clin Pharmacol Ther*, **87**(1):87-92.
233. Schramko AA, Suojaranta-Ylinen RT, Kuitunen AH, Kukkonen SI, Niemi TT: **Rapidly degradable hydroxyethyl starch solutions impair blood coagulation after cardiac surgery: a prospective randomized trial**. *Anesth Analg* 2009, **108**(1):30-36.
234. Segebladh B, Borgstrom A, Nyberg S, Bixo M, Sundstrom-Poromaa I: **Evaluation of different add-back estradiol and progesterone treatments to gonadotropin-releasing hormone agonist treatment in patients with premenstrual dysphoric disorder**. *Am J Obstet Gynecol* 2009, **201**(2):139 e131-138.
235. Seiler CM, Bruckner T, Diener MK, Papyan A, Golcher H, Seidlmayer C, Franck A, Kieser M, Buchler MW, Knaebel HP: **Interrupted or continuous slowly absorbable sutures for closure of primary elective midline abdominal incisions: a multicenter randomized trial (INSECT: ISRCTN24023541)**. *Ann Surg* 2009, **249**(4):576-582.
236. Sen H, Kulahci Y, Bicerer E, Ozkan S, Dagli G, Turan A: **The analgesic effect of paracetamol when added to lidocaine for intravenous regional anesthesia**. *Anesth Analg* 2009, **109**(4):1327-1330.
237. Sen H, Sizlan A, Yanarates O, Emirkadi H, Ozkan S, Dagli G, Turan A: **A comparison of gabapentin and ketamine in acute and chronic pain after hysterectomy**. *Anesth Analg* 2009, **109**(5):1645-1650.
238. Serfaty MA, Haworth D, Blanchard M, Buszewicz M, Murad S, King M: **Clinical effectiveness of individual cognitive behavioral therapy for depressed older people in primary care: a randomized controlled trial**. *Arch Gen Psychiatry* 2009, **66**(12):1332-1340.
239. Setchell KD, Zhao X, Jha P, Heubi JE, Brown NM: **The pharmacokinetic behavior of the soy isoflavone metabolite S-(-)equol and its diastereoisomer R-(+)equol in healthy adults determined by using stable-isotope-labeled tracers**. *Am J Clin Nutr* 2009, **90**(4):1029-1037.
240. Shrivastava VK, Garite TJ, Jenkins SM, Saul L, Rumney P, Preslicka C, Chan K: **A randomized, double-blinded, controlled trial comparing parenteral normal saline with and without dextrose on the course of labor in nulliparas**. *Am J Obstet Gynecol* 2009, **200**(4):379 e371-376.

241. Shyamsundar M, McKeown ST, O'Kane CM, Craig TR, Brown V, Thickett DR, Matthay MA, Taggart CC, Backman JT, Elborn JS *et al*: **Simvastatin decreases lipopolysaccharide-induced pulmonary inflammation in healthy volunteers**. *Am J Respir Crit Care Med* 2009, **179**(12):1107-1114.
242. Siebenhaar F, Degener F, Zuberbier T, Martus P, Maurer M: **High-dose desloratadine decreases wheal volume and improves cold provocation thresholds compared with standard-dose treatment in patients with acquired cold urticaria: a randomized, placebo-controlled, crossover study**. *J Allergy Clin Immunol* 2009, **123**(3):672-679.
243. Silsupadol P, Shumway-Cook A, Lugade V, van Donkelaar P, Chou LS, Mayr U, Woollacott MH: **Effects of single-task versus dual-task training on balance performance in older adults: a double-blind, randomized controlled trial**. *Arch Phys Med Rehabil* 2009, **90**(3):381-387.
244. Siu CW, Lau CP, Lee WL, Lam KF, Tse HF: **Intravenous diltiazem is superior to intravenous amiodarone or digoxin for achieving ventricular rate control in patients with acute uncomplicated atrial fibrillation**. *Crit Care Med* 2009, **37**(7):2174-2179; quiz 2180.
245. Skovbjerg S, Roos K, Holm SE, Grahn Hakansson E, Nowrouzian F, Ivarsson M, Adlerberth I, Wold AE: **Spray bacteriotherapy decreases middle ear fluid in children with secretory otitis media**. *Arch Dis Child* 2009, **94**(2):92-98.
246. Slack D, Nelson L, Patterson D, Burns S, Hakimi K, Robinson L: **The feasibility of hypnotic analgesia in ameliorating pain and anxiety among adults undergoing needle electromyography**. *Am J Phys Med Rehabil* 2009, **88**(1):21-29.
247. Smith SM, Gardner KK, Locke J, Zwart SR: **Vitamin D supplementation during Antarctic winter**. *Am J Clin Nutr* 2009, **89**(4):1092-1098.
248. Smith SS, McCarthy DE, Japuntich SJ, Christiansen B, Piper ME, Jorenby DE, Fraser DL, Fiore MC, Baker TB, Jackson TC: **Comparative effectiveness of 5 smoking cessation pharmacotherapies in primary care clinics**. *Arch Intern Med* 2009, **169**(22):2148-2155.
249. Smolen JS, Kay J, Doyle MK, Landewe R, Matteson EL, Wollenhaupt J, Gaylis N, Murphy FT, Neal JS, Zhou Y *et al*: **Golimumab in patients with active rheumatoid arthritis after treatment with tumour necrosis factor alpha inhibitors (GO-AFTER study): a multicentre, randomised, double-blind, placebo-controlled, phase III trial**. *Lancet* 2009, **374**(9685):210-221.
250. Solomon SD, Appelbaum E, Manning WJ, Verma A, Berglund T, Lukashevich V, Cherif Papst C, Smith BA, Dahlof B: **Effect of the direct Renin inhibitor aliskiren, the Angiotensin receptor blocker losartan, or both on left ventricular mass in patients with hypertension and left ventricular hypertrophy**. *Circulation* 2009, **119**(4):530-537.
251. Song CY, Lin YF, Wei TC, Lin DH, Yen TY, Jan MH: **Surplus value of hip adduction in leg-press exercise in patients with patellofemoral pain syndrome: a randomized controlled trial**. *Phys Ther* 2009, **89**(5):409-418.
252. Songur Y, Senol A, Balkarli A, Basturk A, Cerci S: **Triple or quadruple tetracycline-based therapies versus standard triple treatment for Helicobacter pylori treatment**. *Am J Med Sci* 2009, **338**(1):50-53.
253. Strengell T, Uhari M, Tarkka R, Uusimaa J, Alen R, Lautala P, Rantala H: **Antipyretic agents for preventing recurrences of febrile seizures: randomized controlled trial**. *Arch Pediatr Adolesc Med* 2009, **163**(9):799-804.

254. Szymlek-Gay EA, Ferguson EL, Heath AL, Gray AR, Gibson RS: **Food-based strategies improve iron status in toddlers: a randomized controlled trial12**. *Am J Clin Nutr* 2009, **90**(6):1541-1551.
255. Tack J, van Outryve M, Beyens G, Kerstens R, Vandeplassche L: **Prucalopride (Resolor) in the treatment of severe chronic constipation in patients dissatisfied with laxatives**. *Gut* 2009, **58**(3):357-365.
256. Taghavi SA, Jafari A, Eshraghian A: **Efficacy of a new therapeutic regimen versus two routinely prescribed treatments for eradication of Helicobacter pylori: a randomized, double-blind study of doxycycline, co-amoxiclav, and omeprazole in Iranian patients**. *Dig Dis Sci* 2009, **54**(3):599-603.
257. Talab HF, Zabani IA, Abdelrahman HS, Bukhari WL, Mamoun I, Ashour MA, Sadeq BB, El Sayed SI: **Intraoperative ventilatory strategies for prevention of pulmonary atelectasis in obese patients undergoing laparoscopic bariatric surgery**. *Anesth Analg* 2009, **109**(5):1511-1516.
258. Tardy AL, Lambert-Porcheron S, Malpuech-Brugere C, Giraudet C, Rigaudiere JP, Laillet B, Leruyet P, Peyraud JL, Boirie Y, Laville M *et al*: **Dairy and industrial sources of trans fat do not impair peripheral insulin sensitivity in overweight women**. *Am J Clin Nutr* 2009, **90**(1):88-94.
259. Tarhini AA, Millward M, Mainwaring P, Kefford R, Logan T, Pavlick A, Kathman SJ, Laubscher KH, Dar MM, Kirkwood JM: **A phase 2, randomized study of SB-485232, rhIL-18, in patients with previously untreated metastatic melanoma**. *Cancer* 2009, **115**(4):859-868.
260. Teerlink JR, Metra M, Felker GM, Ponikowski P, Voors AA, Weatherley BD, Marmor A, Katz A, Grzybowski J, Unemori E *et al*: **Relaxin for the treatment of patients with acute heart failure (Pre-RELAX-AHF): a multicentre, randomised, placebo-controlled, parallel-group, dose-finding phase IIb study**. *Lancet* 2009, **373**(9673):1429-1439.
261. Thomson CD, Campbell JM, Miller J, Skeaff SA, Livingstone V: **Selenium and iodine supplementation: effect on thyroid function of older New Zealanders**. *Am J Clin Nutr* 2009, **90**(4):1038-1046.
262. Timsit JF, Schwebel C, Bouadma L, Geffroy A, Garrouste-Orgeas M, Pease S, Herault MC, Haouache H, Calvino-Gunther S, Gestin B *et al*: **Chlorhexidine-impregnated sponges and less frequent dressing changes for prevention of catheter-related infections in critically ill adults: a randomized controlled trial**. *Jama* 2009, **301**(12):1231-1241.
263. Toledo P, McCarthy RJ, Ebarvia MJ, Huser CJ, Wong CA: **The interaction between epidural 2-chloroprocaine and morphine: a randomized controlled trial of the effect of drug administration timing on the efficacy of morphine analgesia**. *Anesth Analg* 2009, **109**(1):168-173.
264. Tuomikoski P, Ebert P, Groop PH, Haapalahti P, Hautamaki H, Ronnback M, Ylikorkala O, Mikkola TS: **Effect of hot flushes on vascular function: a randomized controlled trial**. *Obstet Gynecol* 2009, **114**(4):777-785.
265. Tyson SF, Rogerson L: **Assistive walking devices in nonambulant patients undergoing rehabilitation after stroke: the effects on functional mobility, walking impairments, and patients' opinion**. *Arch Phys Med Rehabil* 2009, **90**(3):475-479.
266. van Dommelen P, Kamphuis M, van Leerdam FJ, de Wilde JA, Rijpstra A, Campagne AE, Verkerk PH: **The short- and long-term effects of simple behavioral interventions for nocturnal enuresis in young children: a randomized controlled trial**. *J Pediatr* 2009, **154**(5):662-666.

267. van Gils EJ, Veenhoven RH, Hak E, Rodenburg GD, Bogaert D, Ijzerman EP, Bruin JP, van Alphen L, Sanders EA: **Effect of reduced-dose schedules with 7-valent pneumococcal conjugate vaccine on nasopharyngeal pneumococcal carriage in children: a randomized controlled trial**. *Jama* 2009, **302**(2):159-167.
268. van Zundert A, Maassen R, Lee R, Willems R, Timmerman M, Siemonsma M, Buise M, Wiepking M: **A Macintosh laryngoscope blade for videolaryngoscopy reduces stylet use in patients with normal airways**. *Anesth Analg* 2009, **109**(3):825-831.
269. Vargas F, Thille A, Lyazidi A, Campo FR, Brochard L: **Helmet with specific settings versus facemask for noninvasive ventilation**. *Crit Care Med* 2009, **37**(6):1921-1928.
270. Veselis RA, Pryor KO, Reinsel RA, Li Y, Mehta M, Johnson R, Jr.: **Propofol and midazolam inhibit conscious memory processes very soon after encoding: an event-related potential study of familiarity and recollection in volunteers**. *Anesthesiology* 2009, **110**(2):295-312.
271. Vogler CM, Sherrington C, Ogle SJ, Lord SR: **Reducing risk of falling in older people discharged from hospital: a randomized controlled trial comparing seated exercises, weight-bearing exercises, and social visits**. *Arch Phys Med Rehabil* 2009, **90**(8):1317-1324.
272. von Goedecke A, Mitterschiffthaler L, Paal P, Mitterlechner T, Wenzel V, Herff H: **Optimising the unprotected airway with a prototype Jaw-Thrust-Device--a prospective randomised cross-over study**. *Anaesthesia* 2009, **64**(11):1236-1240.
273. von Hertzen H, Piaggio G, Wojdyla D, Marions L, My Huong NT, Tang OS, Fang AH, Wu SC, Kalmar L, Mittal S *et al*: **Two mifepristone doses and two intervals of misoprostol administration for termination of early pregnancy: a randomised factorial controlled equivalence trial**. *Bjog* 2009, **116**(3):381-389.
274. Vona M, Codeluppi GM, Iannino T, Ferrari E, Bogousslavsky J, von Segesser LK: **Effects of different types of exercise training followed by detraining on endothelium-dependent dilation in patients with recent myocardial infarction**. *Circulation* 2009, **119**(12):1601-1608.
275. Wallace DJ, Stohl W, Furie RA, Lisse JR, McKay JD, Merrill JT, Petri MA, Ginzler EM, Chatham WW, McCune WJ *et al*: **A phase II, randomized, double-blind, placebo-controlled, dose-ranging study of belimumab in patients with active systemic lupus erythematosus**. *Arthritis Rheum* 2009, **61**(9):1168-1178.
276. Wang SM, Dezinno P, Lin EC, Lin H, Yue JJ, Berman MR, Braveman F, Kain ZN: **Auricular acupuncture as a treatment for pregnant women who have low back and posterior pelvic pain: a pilot study**. *Am J Obstet Gynecol* 2009, **201**(3):271 e271-279.
277. Weaver CM, Martin BR, Jackson GS, McCabe GP, Nolan JR, McCabe LD, Barnes S, Reinwald S, Boris ME, Peacock M: **Antiresorptive effects of phytoestrogen supplements compared with estradiol or risedronate in postmenopausal women using (41)Ca methodology**. *J Clin Endocrinol Metab* 2009, **94**(10):3798-3805.
278. Weber JS, Zarour H, Redman B, Trefzer U, O'Day S, van den Eertwegh AJ, Marshall E, Wagner S: **Randomized phase 2/3 trial of CpG oligodeoxynucleotide PF-3512676 alone or with dacarbazine for patients with unresectable stage III and IV melanoma**. *Cancer* 2009, **115**(17):3944-3954.
279. Weber MA, Black H, Bakris G, Krum H, Linas S, Weiss R, Linseman JV, Wiens BL, Warren MS, Lindholm LH: **A selective endothelin-receptor antagonist to reduce blood pressure in patients with treatment-resistant hypertension: a randomised, double-blind, placebo-controlled trial**. *Lancet* 2009, **374**(9699):1423-1431.
280. Wei DH, Raizman NM, Bottino CJ, Jobin CM, Strauch RJ, Rosenwasser MP: **Unstable distal radial fractures treated with external fixation, a radial column**

plate, or a volar plate. A prospective randomized trial. *J Bone Joint Surg Am* 2009, **91**(7):1568-1577.

281. Wen RJ, Leslie K, Rajendra P: **Pre-operative forced-air warming as a method of anxiolysis**. *Anaesthesia* 2009, **64**(10):1077-1080.
282. Wenzel SE, Barnes PJ, Bleecker ER, Bousquet J, Busse W, Dahlen SE, Holgate ST, Meyers DA, Rabe KF, Antczak A *et al*: **A randomized, double-blind, placebo-controlled study of tumor necrosis factor-alpha blockade in severe persistent asthma**. *Am J Respir Crit Care Med* 2009, **179**(7):549-558.
283. White DK, Wagenaar RC, Ellis TD, Tickle-Degnen L: **Changes in walking activity and endurance following rehabilitation for people with Parkinson disease**. *Arch Phys Med Rehabil* 2009, **90**(1):43-50.
284. White PF, Tufanogullari B, Taylor J, Klein K: **The effect of pregabalin on preoperative anxiety and sedation levels: a dose-ranging study**. *Anesth Analg* 2009, **108**(4):1140-1145.
285. White WB, Schnitzer TJ, Fleming R, Duquesroix B, Beekman M: **Effects of the cyclooxygenase inhibiting nitric oxide donator naproxcinod versus naproxen on systemic blood pressure in patients with osteoarthritis**. *Am J Cardiol* 2009, **104**(6):840-845.
286. Wise RA, Bartlett SJ, Brown ED, Castro M, Cohen R, Holbrook JT, Irvin CG, Rand CS, Sockrider MM, Sugar EA: **Randomized trial of the effect of drug presentation on asthma outcomes: the American Lung Association Asthma Clinical Research Centers**. *J Allergy Clin Immunol* 2009, **124**(3):436-444, 444e431-438.
287. Worthley DL, Le Leu RK, Whitehall VL, Conlon M, Christophersen C, Belobrajdic D, Mallitt KA, Hu Y, Irahara N, Ogino S *et al*: **A human, double-blind, placebo-controlled, crossover trial of prebiotic, probiotic, and synbiotic supplementation: effects on luminal, inflammatory, epigenetic, and epithelial biomarkers of colorectal cancer**. *Am J Clin Nutr* 2009, **90**(3):578-586.
288. Wroblewski JJ, Wells JA, 3rd, Adamis AP, Buggage RR, Cunningham ET, Jr., Goldbaum M, Guyer DR, Katz B, Altaweel MM: **Pegaptanib sodium for macular edema secondary to central retinal vein occlusion**. *Arch Ophthalmol* 2009, **127**(4):374-380.
289. Wu HG, Song SY, Kim YS, Oh YT, Lee CG, Keum KC, Ahn YC, Lee SW: **Therapeutic effect of recombinant human epidermal growth factor (RhEGF) on mucositis in patients undergoing radiotherapy, with or without chemotherapy, for head and neck cancer: a double-blind placebo-controlled prospective phase 2 multi-institutional clinical trial**. *Cancer* 2009, **115**(16):3699-3708.
290. Yamakage M, Iwasaki S, Jeong SW, Satoh J, Namiki A: **Beta-1 selective adrenergic antagonist landiolol and esmolol can be safely used in patients with airway hyperreactivity**. *Heart Lung* 2009, **38**(1):48-55.
291. Yao YS, Qian B, Chen BZ, Wang R, Tan L: **The optimum concentration of levobupivacaine for intra-operative caudal analgesia in children undergoing inguinal hernia repair at equal volumes of injectate**. *Anaesthesia* 2009, **64**(1):23-26.
292. Yeager MP, Rassias AJ, Pioli PA, Beach ML, Wardwell K, Collins JE, Lee HK, Guyre PM: **Pretreatment with stress cortisol enhances the human systemic inflammatory response to bacterial endotoxin**. *Crit Care Med* 2009, **37**(10):2727-2732.
293. Yoon SH, Rah UW, Sheen SS, Cho KH: **Comparison of 3 needle sizes for trigger point injection in myofascial pain syndrome of upper- and middle-trapezius**

muscle: a randomized controlled trial. *Arch Phys Med Rehabil* 2009, **90**(8):1332-1339.
294. Yusuf S, Pais P, Afzal R, Xavier D, Teo K, Eikelboom J, Sigamani A, Mohan V, Gupta R, Thomas N: **Effects of a polypill (Polycap) on risk factors in middle-aged individuals without cardiovascular disease (TIPS): a phase II, double-blind, randomised trial**. *Lancet* 2009, **373**(9672):1341-1351.
295. Ziegler EE, Nelson SE, Jeter JM: **Iron status of breastfed infants is improved equally by medicinal iron and iron-fortified cereal**. *Am J Clin Nutr* 2009, **90**(1):76-87.
296. Zijlstra N, de Wijk RA, Mars M, Stafleu A, de Graaf C: **Effect of bite size and oral processing time of a semisolid food on satiation**. *Am J Clin Nutr* 2009, **90**(2):269-275.
297. Zorko N, Kamenik M, Starc V: **The effect of Trendelenburg position, lactated Ringer's solution and 6% hydroxyethyl starch solution on cardiac output after spinal anesthesia**. *Anesth Analg* 2009, **108**(2):655-659.
298. Zygun DA, Nortje J, Hutchinson PJ, Timofeev I, Menon DK, Gupta AK: **The effect of red blood cell transfusion on cerebral oxygenation and metabolism after severe traumatic brain injury**. *Crit Care Med* 2009, **37**(3):1074-1078.
